# *Vaccinium* Species—Unexplored Sources of Active Constituents for Cosmeceuticals

**DOI:** 10.3390/biom14091110

**Published:** 2024-09-03

**Authors:** Wirginia Kukula-Koch, Natalia Dycha, Paulina Lechwar, Magdalena Lasota, Estera Okoń, Paweł Szczeblewski, Anna Wawruszak, Dominik Tarabasz, Jane Hubert, Piotr Wilkołek, Maria Halabalaki, Katarzyna Gaweł-Bęben

**Affiliations:** 1 Department of Pharmacognosy with Medicinal Plants Garden, Medical University of Lublin, 1 Chodzki Str., 20-093 Lublin, Poland; virginia.kukula@gmail.com (W.K.-K.); dychanatalia98@gmail.com (N.D.); dominikanes@o2.pl (D.T.); 2 Department of Cosmetology, The University of Information Technology and Management in Rzeszow, Sucharskiego 2, 35-225 Rzeszow, Poland; plechwar@wsiz.edu.pl (P.L.); mlasota@wsiz.edu.pl (M.L.); kagawel@wsiz.edu.pl (K.G.-B.); 3 Department of Biochemistry and Molecular Biology, Medical University of Lublin, 1 Chodzki Str., 20-093 Lublin, Poland; estera.okon@umlub.pl (E.O.); anna.wawruszak@umlub.pl (A.W.); 4 Department of Pharmaceutical Technology and Biochemistry and BioTechMed Centre, Faculty of Chemistry, Gdansk University of Technology, Gabriela Narutowicza Str. 11/12, 80-233 Gdansk, Poland; pawel.szczeblewski@pg.edu.pl; 5 NatExplore, 51140 Prouilly, France; jane.hubert@nat-explore.com; 6 Department of Clinical Diagnostics and Veterinary Dermatology, University of Life Sciences in Lublin, 32 Gleboka Str., 20-612 Lublin, Poland; 7 Division of Pharmacognosy and Natural Products Chemistry, Department of Pharmacy, National and Kapodistrian University of Athens, Panepistimioupoli Zografou, 15771 Athens, Greece

**Keywords:** *Vaccinium* spp., Ericaceae, cosmetics, berries, UV radiation, enzyme inhibitory properties, antioxidants, natural products

## Abstract

The genus *Vaccinium* is represented by shrubs growing in a temperate climate that have been used for ages as traditional remedies in the treatment of digestive problems, in diabetes, renal stones or as antiseptics due to the presence of polyphenols (anthocyanins, flavonoids and tannins) in their fruits and leaves. Recent studies confirm their marked potential in the treatment of skin disorders and as skin care cosmetics. The aim of this review is to present the role of *Vaccinium* spp. as cosmetic products, highlight their potential and prove the biological properties exerted by the extracts from different species that can be useful for the preparation of innovative cosmetics. In the manuscript both skin care and therapeutic applications of the representatives of this gender will be discussed that include the antioxidant, skin lightening, UV-protective, antimicrobial, anti-inflammatory, and chemopreventive properties to shed new light on these underestimated plants.

## 1. Introduction

The genus *Vaccinium* L. (Ericaceae) includes more than 450 species of shrubs or dwarf shrubs, growing mainly in cooler areas of the northern hemisphere, including Europe, North and Central America, Asia and South and Central Africa. The European flora comprises *V. oxycoccos* (common cranberry), *V. corymbosum* (blueberry), *V. vitis-idaea* (lingonberry) *V. microcarpum* (small bog cranberry), *V. macrocarpon* (American cranberry), *V. uliginosum* (bog bilberry), *V. myrtillus* (bilberry), *V. arctostaphylos* (Caucasian whortleberry), and *V. cylindraceum* (Azores blueberry). *V. corymbosum* (northern highbush blueberry) is a North American species widely cultivated in Europe for its big edible fruits [1,2]. *Vaccinium* species have been used in traditional medicine of different cultures and the berries are widely consumed as food. The highest economic value belong to the Vaccinioidae subfamily, represented by cranberry, blueberry, huckleberry, and bilberry [3].

*Vaccinium* species have a long history of use in traditional European medicine. Fruits and leaves of *V. myrtillus* and *V. vitis-idaea* has been traditionally used as a remedy for coughs and fever, for the treatment of renal stones, intestinal and liver disorders, and diabetes, and for their astringent, tonic, and antiseptic properties [4,5]. Leaves and fruits of *V. arctostaphylos* are known for their anti-hypertensive and anti-diabetic properties [6] and the fruits of *V. corymbosum* have been used as natural therapy for diabetes, inflammation and gastrointestinal disorders [7,8]. Traditional uses of *Vaccinium*-based preparations have been supported by modern research and for many *Vaccinium* components, such as polyphenols, anthocyanins and flavonoids, important biological functions have been described in scientific literature. *Vaccinium* extracts and single phytochemicals are known mainly for their antioxidant, anti-inflammatory, antidiabetic and endothelium protective activities [1,9]. Other studies implicated its effectiveness in the treatment of cardiovascular and neurodegenerative diseases, atherosclerosis and rheumatoid arthritis [2]. It is also considered that *Vaccinium* fruits, particularly those with high content of anthocyanins, are able to initiate apoptosis and delay proliferation and metastasis of cancer cells [1,2,10,11].

Bioactive substances contained in *Vaccinium* leaves and berries can also have health benefits when applied to the skin, which is why *Vaccinium*-based ingredients are becoming more and more popular in modern cosmetology and dermatology. Topical usage of *Vaccinium* ingredients is supported also by its traditional uses. *V. myrtillus* fruit extract has been traditionally used on the skin due to its antiseptic, astringent and tonic properties [12]. Extracts obtained from berries and leaves of *V. vitis-idaea* are known to accelerate wound healing [13].

The health-beneficial properties of the *Vaccinium* genus have been summarized in many review articles published in recent years [1,2,9,14]. However, there is no publication summarizing the current cosmetic and dermatological use of the genus *Vaccinium* as well as highlighting its skin-beneficial potential. Therefore, the present review is designed to report the current knowledge on the cosmetic and dermatological properties of plant species that belong to the *Vaccinium* genus, their phytochemicals, with particular emphasis on their anti-aging, skin-lightening, anti-inflammatory, UV-protecting and anti-microbial properties.

## 2. Phytochemical Composition

The traditional phytotherapeutic applications of *Vaccinium* species are strictly related to a rich representation of polyphenols in their extracts that joins the presence of simple phenolic acids, flavonoids, flavonoid glycosides, anthocyanins (ACNs) and complex tannins. Their content is considerably affected by the stage of development, the geographical localization of the plant, by the genetic causes and the amount of sunlight [9].

The most precious group of metabolites extracted from *Vaccinium* spp. (blueberry, cranberry, bilberry, and lingonberry) are ACNs such as cyanidin, peonidin, petunidin, delphinidin, malvidin and their glucosides. Also flavonols like quercetin, kaempferol, myricetin and flavanols like epicatechin that were proved to exhibit various pharmacological actions were previously described as the leading constituents of this gender [15,16].

**Figure 1:**
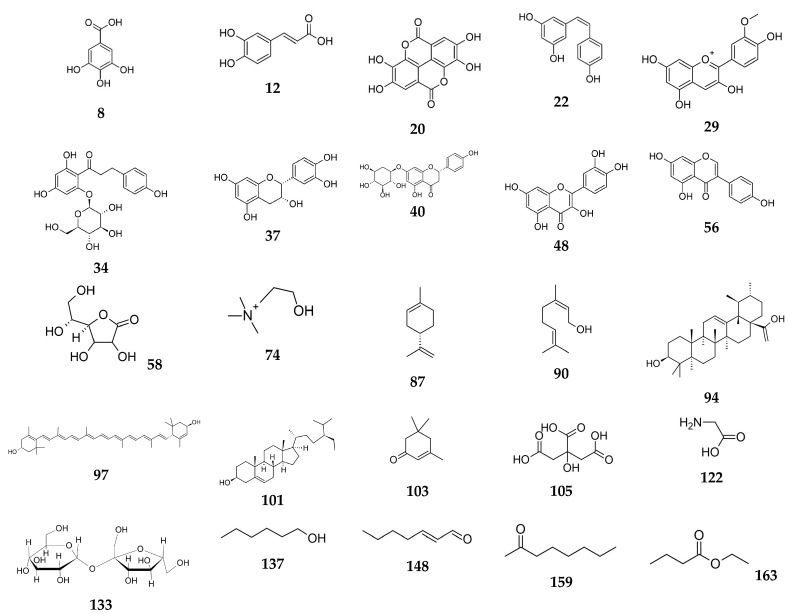
Chemical structures of the major representatives of each class of metabolites distributed in *Vaccinium* species.

The list of metabolites reported in *Vaccinium* species is very long. In this review paper a detailed literature survey on the well-investigated species, namely *Vaccinium macroparpon*, is presented (Table 1, Figure 1) and treated as a representative plant with a proven diverse composition due to its vast industrial applications. As mentioned by the scientific literature, 500 million tons of cranberries are produced annually, out of which only 5% stay in an unprocessed form. Cranberry juice is one of the most important industrial products with a variety of applications, including pharma, food, cosmetic, animal feed, and soil fertilizing industries [17].

As presented below, *V. macrocarpon* is proved to contain metabolites that belong to phenolic acids, stilbenes, flavonoids (anthocyanidins, chalcons, flavonols, flavanols, flavanones, and isoflavonoids), vitamins, fatty acids, esters of fatty acids, sterols, oxygenated hydrocarbons, terpenoids, organic acids, aminoacids, and others).

The multitude of the components presented below stand for a unique fingerprint of *Vaccinium* species and confirm their ability to exhibit a wide range of biological properties with high safety of administration.

The structures of the selected metabolites—the representatives of each group of natural products presented in the Table 1 are shown in the Figure 1 below.

## 3. *Vaccinium* Species in Skin Care

*Vaccinium* species have been used in traditional medicine due to their vast therapeutic applications and pharmacological actions. Nowadays they are present in the market in the form of dried fruits, powdered plant material or dried extracts. The type of raw material determines how it is used. For example, fresh fruits of *Vaccinium myrtillus* is used as anthocyanin-containing substance, whereas dried fruit due to a higher relative tannin content is administered in diarrhoeas. Various extracts from *Vaccinium* spp. are registered in the CosIng database, in a database that is a source of information about the available cosmetic ingredients which can be used for the production of cosmetics and cosmeceutics [20].

Table 2 lists the registered records that include different species of *Vaccinium*, their different organs and types of extracts together with the awaited functions in cosmetic products. As can be seen below, the list of records is high proving a ubiquity of this group of plants. Among the listed properties of berries skin-conditioning, abrasive, astringent, moisturising and antioxidant properties should be underlined.

In the following sections detailed information about the proved biological properties of different *Vaccinium* spp. are described together with the clarification of the potential mechanisms of action and active doses.

### 3.1. Skin-Lightening Properties

Melanin synthesis (melanogenesis) in an important mechanism, protecting the skin from the harmful influence of ultraviolet radiation. However, an increased melanin synthesis or its uneven distribution in the epidermis results in one of the most common aesthetic disorders, called hyperpigmentation. Therefore, cosmetic products and active ingredients reducing melanin biosynthesis are constantly one of the most important features of the global cosmetic market [21]. Main target of skin-lightening ingredients is tyrosinase (EC 1.14.18.1), also known as a polyphenol oxidase. It is a copper-containing enzyme responsible for the catalysis of the first two rate-limiting steps of melanin synthesis: the hydroxylation of L-tyrosine by monophenolase activity (monophenol L-tyrosine to diphenol 3,4-dihydroxyphenylalanine, L-DOPA), and oxidation of L-DOPA to the *o*-dopaquinone (diphenolase activity) [22]. Tyrosinase inhibition and regulation of melanogenesis have also been proved for extracts and active compounds obtained from several *Vaccinium* species.

Commercially manufactured juices from the fruits of cranberry (*Vaccinium macrocarpon*) and blueberry (*Vaccinium myrtillus*) showed a dose-dependent tyrosinase inhibitory potential. At the highest concentration of 1 mg/mL, blueberry juice inhibited tyrosinase activity by 78%, which is a similar result to the kojic acid at the final concentration of 0.02 mg/mL. Cranberry juice at the concentration of 1 mg/mL showed a 58% inhibition of tyrosinase. For all tested concentrations, blueberry juice was characterized by a better ability to reduce tyrosinase activity, which represents a potential use in skin lightening cosmetics. The IC_50_ values calculated for tyrosinase inhibition method were 0.1064 ± 0.03630 µg/mL for blueberry juice and 0.4814 ± 0.09839 µg/mL for cranberry juice. The particular compounds responsible for observed anti-tyrosinase effect have not been identified, but both juices were rich in total polyphenols (27.44 ± 4.892 μg gallic acid equivalents, GAE, per 1 mg of blueberry juice and 23.76 ± 1.407 μg GAE per mg of cranberry juice) and anthocyanins (3.909 mg per mg of blueberry juice and 0.398 mg per mg of cranberry juice) [23].

In another study Studzińska-Sroka and others examined tyrosinase inhibitory effect of extracts from *Vaccinium myrtillus* (bilberry) and *Vaccinium corymbosum* (blueberry) in four different solvents: water, methanol, and mixtures of methanol–water 1:1 *v*/*v*, or acetone–water 1:1 *v*/*v*. The presented results proved that all *V. myrtillus* extracts exhibited tyrosinase inhibition, with the acetone-water extract being the most active (ca. 45% tyrosinase inhibition at the tested extract concentration of 0.25 mg/mL) from all samples. Among tested *V. corymbosum* extracts only the methanol-water and acetone-water extracts were inhibiting tyrosinase, with ca. 20% and 45% inhibition at 0.25 mg/mL, respectively. The phytochemical composition of the most active extracts has not been investigated in details but they both contained significant amounts of total polyphenols (18.43 ± 0.90 and 28.17 ± 1.03 mg GAE/g od dried extract, respectively), total flavonoids (0.488 ± 0.01 and 0.321 ± 0.02 mg quercetin equivalents, QE, per g of dried extract, respectively) and anthocyanins (0.27 ± 0.02 and 0.24 ± 0.03% cyanidin 3-glucoside, respectively). The most active acetone-water extract from *Vaccinium corymbosum* L. was introduced as an active ingredient of chitosan-based hydrogels at different proportions: as 0.5% of extract and 2% of chitosan (H1), as 1% of extract and 2% of chitosan (H2), as 0.5% of extract and 3% of chitosan (H3), and as 1% of extract and 3% of chitosan (H4). Interestingly, the tyrosinase inhibitory activity increased together with an increasing concentration of chitosan and a decreasing content of *V. corymbosum* extract [24]. The authors explained this observation using the literature data, showing that chitosan is able to bind the cooper ion located in the active site of tyrosinase, and therefore it altered its catalytic activity [25].

All of the tyrosinase inhibitory activities described above were shown using a commercially available mushroom tyrosinase, widely used in cosmetic-related research. However, effective mushroom tyrosinase inhibitors are sometimes not active against mammalian enzyme [26]. Kim et al. identified the extract from the aerial parts of *V. bracteatum* as an effective inhibitor of human tyrosinase, obtained from the lysate of stably transfected HEK293 cells. Interestingly, this extract showed no inhibitory activity towards commercially available mushroom tyrosinase. Fractionation of the extract identified *p*-coumaric acid (PCA) as the main active constituent responsible for tyrosinase inhibitory potential of the extract. The influence of PCA on melanogenesis was further confirmed in vitro, using a reconstructed human epidermis model with melanocytes, namely MelanoDerm. The PCA concentration of 5 mM decreased the melanin content by 12.7%, whereas a positive control with the melanogenesis inhibitor—arbutin (5 mM) decreased melanin level by only 7.6% [27].

Yang and co-workers investigated tyrosinase inhibitory potential of leaf extracts obtained from *Vaccinium dunalianum* by subcritical water extraction (SWE extract) or hot water extraction (HWE extract). SWE and HWE extracts showed significant inhibitory activity on monophenolase activity of tyrosinase when used in a concentration range of 0.005–0.25 mg/mL. HWE had lower inhibition activity than SWE at lower concentrations. The IC_50_ values for SWE and HWE were 11.81 ± 0.52 μg/mL and 24.50 ± 1.78 μg/mL, respectively. On the other hand, the IC_50_ values of kojic acid in respect of a monophenolase inhibition was 9.33 ± 0.66 μg/mL. Interestingly, SWE showed higher diphenolase inhibitory activity compared to HWE and kojic acid, that was 21.17 ± 1.83 μg/mL, while of the HWE’s it was calculated as 86.98 ± 3.46 μg/mL (kojic acid IC_50_ = 76.63 ± 4.71 μg/mL) [28]. In another research by the same group 6′-*O*-caffeoylarbutin, β-arbutin, and chlorogenic acid were identified as the major polyphenols responsible for the monophenolase and diphenolase inhibitory activity of *V. dunalianum* leaf extract [29]. The skin lightening potential of 6′-*O*-caffeoylarbutin was compared with arbutin using zebrafish melanogenesis assay. The 6′-*O*-caffeoylarbutin was characterized by a stronger tyrosinase inhibitory effect on melanin formation compared with arbutin at the same concentrations. As a result, the IC_20_ values for 6′-*O*-caffeoylarbutin and arbutin were 63.89 µM and 244.6 µM, respectively. The lowest level required to prevent melanin production in zebrafish was 60 µM for 6′-*O*-caffeoylarbutin and 100 µM for arbutin. Zebrafish model was also used in this study to compare the toxicity of both compounds in varying concentrations ranging from 10 µM to 3 mM. No mortalities were observed at the highest concentration of 3 mM after 52 hours post-fertilization for both compounds. By 96 hours post-fertilization, the selected non-toxic concentrations of 6′-*O*-caffeoylarbutin and arbutin were 2 mM and 1 mM, respectively. These findings suggest that the derivative of caffeic acid expressed greater safety margins compared to arbutin itself. Interestingly, additional studies have shown that melanin synthesis can be restored after the removal of 6′-*O*-caffeoylarbutin from the wells [30].

Another active compound with promising skin lightening potential identified in various *Vaccinium* berries is pterostilbene [31]. Pterostilbene showed a potent inhibitory effect on melanogenesis in B16F10 cells (at 3 μM) in both in vitro human skin models (at 10 μM) and in zebrafish embryos (at 3 μM). Besides, pterostilbene not only inhibited melanogenesis, but also inhibited melanocyte dendritic development and melanosome transport. Pterostilbene mainly plays a role by inhibiting cAMP/PKA/CREB signal pathway. After the cAMP/PKA/CREB signaling pathway was inhibited, tyrosinase activity and the expression of tyrosinase, MITF, and other factors regulating melanogenesis: Rab27A, Rab17 and gp100, which in turn suppressed melanogenesis, melanocyte dendritic development and melanosome transport [32].

The abundant information on the activity of *Vaccinium* species in the inhibition of hyperpigmentation process (see Table 3) confirm the presence of several constituents that can influence the action of the total extract. The whitening properties of the shrubs was confirmed using different hyperpigmentation models, that included the spectrophotometric studies in mushroom and murine tyrosinases, but also tests in cells with an increased melanogenesis or zebrafish based assays. Phenolic compounds present in the extracts of different berries were proved to show this particular action—when present in the mixtures or when isolated from the extracts, with high safety premises.

### 3.2. Anti-Aging Properties of Vaccinium spp.

*Vaccinium* species were proved to exhibit interesting anti-enzymatic properties and show a potential to be included in anti-aging cosmetics. One of the potential targets of berry metabolites is collagen. Type I collagen is the most common type of collagen found in the body that is spread, among others, in the skin, subcutaneous tissue, scar tissue or in tendons [33].

In the research work of Vu Dang La and co-investigators cranberry proanthocyanidins (AC-PAC) were isolated from *Vaccinium macrocarpon* fruits using solid phase chromatography and their effect on type I collagen was studied by extracellular proteinases produced by *P. gingivalis*. According to the authors [34] all concentrations of AC-PAC significantly inhibited collagen degradation. Among the tested doses, the concentration of 100 µg/mL AC-PAC showed the highest inhibitory effect—88.7 ± 8.5% [35].

In the research work entitled “Anti-Aging Properties of Chitosan-Based Hydrogels Rich in Bilberry Fruit Extract”, two *Vaccinium* species, namely *V. myrtillus* and *V. corymbosum* were assessed concerning their hyaluronidase inhibitory properties [24]. It was proven that the extracts with the most significant hyaluronidase inhibition effect, regardless of the tested species, were the water-acetone extracts that were capable to inhibit more than 90% of the enzyme’s potential at 0.05 g/mL. Also, according to the authors the hyaluronidase inhibitory effect increased with an increasing concentration of chitosan and a decreasing content of the extract when preparing ready formulations, in accordance with the previously described tyrosinase inhibitory action. The IC_50_ values of the hydrogel containing 0.5% extract and 3.0% chitosan was 116.26 ± 26.65 [mg hydrogel]. As discussed above the analyzed extracts were rich sources of polyphenols, flavonoids and chlorogenic acid that could determine their total activity [24].

Pageon and co-investigators tested the blueberry extract (*Vaccinium angustifolium*) for the inhibition of glycation process. Glycation is a phenomenon related to the aging process and is observed as a slow, non-enzymatic reaction between free amino groups in proteins. The final steps of this reaction lead to the formation of advanced glycation end products (AGEs) that change the structure and propertied of the skin. In this case during natural aging or in photoaging changed collagen properties and triggered fibroblasts’ dysfunctions and elevated reactive oxygen species are observed. In the mentioned study the glycation of bovine type I collagen using D-ribose with/without amino acid-anidine blueberry extract at a concentration of 5% decreased the fluorescence of the collagen demonstrating an inhibitory effect towards protein glycation by 76% [36,37].

The results of the antiaging properties from in vitro studies were confirmed also in more complicated models, for instance on an in vitro human skin model that was reconstructed by seeding human epidermal keratinocytes on top of skin counterparts using stainless steel rings. After adding 5% blueberry extract, aminoguanidine, ribose and ribose extract and amino-guanidine with ribose, immunostaining of carboxymethyl lysine (CML) and β1 integrin was performed using mouse monoclonal antibodies (Mabs) and the level of MMP-1 content was measured using an enzyme-linked immunosorbent assay. As a result, strong immunostaining was obtained on ribose-treated collagen using the anti-CML antibody which was reverted in samples treated with blueberry extract, indicating its glycation inhibition properties. When immunostaining for β1 integrin, the sample with extract and ribose showed a return to the pattern of the control group (extract), which also indicates an anti-glycation effect. Furthermore, blueberry extract reduced the amount of MMP-1 that was induced by ribose in the reconstructed skin medium [37].

In another study the wrinkle formation was assessed on normal and UVB-irradiated mouse skin samples. The analysis included the total wrinkle surface (mm^2^), average wrinkle depth (mm), number of wrinkles, and average wrinkle length (mm) measurements. Mice were given a low (EL), medium (EM) and high (EH) dose of *Vaccinium uliginosum* extract enriched with anthocyanins, and then were irradiated with UVB. Compared to the control, the best anti-wrinkle effect was observed with the highest dose, and for this group the total area of wrinkles was 4 times smaller, the number of wrinkles was 1/3 smaller, the average length of wrinkles was about 37.5% smaller, the depth of wrinkles was 50% lower, and the average depth of wrinkles decreased by approximately 36% [10].

The scientific literature shows an example of a clinical study on healthy women aged 35–50 who took part in a randomized, double-blind, placebo-controlled clinical trial. The effect of lingonberry and amla fruit extracts administered in the form of a drink on the condition of the skin proved the elasticity and thickness restoration. The most beneficial effects were demonstrated for the extract prepared from 50 mg of dry lingonberry extract and 60 mg of dry amla fruit extract (LAE double) after 12 weeks of use. The elasticity of the skin on the cheek increased up to two times when measured with a 2 mm probe (superficial part of the skin), and up to five times when measured with a 6 mm probe (deep part of the skin). The skin thickness increased by 120 µm as compared to the control [38].

The collected information, summarized in the Table 4, proves multidirectional activities of *Vaccinium* extracts in terms of their antiaging potential. Berries were proved to influence the synthesis of collagen I, decrease the glycation of proteins that occurs within skin aging, increase the thickness of skin, decrease the wrinkles formation and inhibit the decomposition of hyaluronic acid in the skin by affecting the hyaluronidase enzyme. The observed properties stand for an multifunctional role of berries that can be used not only in cosmetics, but also in superfoods, pharma and supplements.

### 3.3. Antioxidant Activity

Plants of the *Vaccinium* genus are exceptionally rich in antioxidant compounds, making them valuable species in cosmetology that can be used for skin care and anti-aging prevention. The extracts were proved to contain abundant quantities of polyphenolic compounds such as flavonoids, phenolic acids and anthocyanins, which are attributed the strongest antiradical molecules, and also marked content of ascorbic acid or tannins, whose concentration varies depending on the species and its maturity [1]. Among the flavonoids distributed in the genus, flavonols constitute an important group. Among them quercetin, myricetin and their glycosidic derivatives account for up to 30%. Anthocyanins, including procyanidins, anthocyanidins (cyanidin, malvidin, peonidin, delphinidin, petunidin), can reach up to 24% of all polyphenolic compounds. Phenolic acids, which primarily include *p*-coumaric acid, chlorogenic acid, caffeic acid, ferulic acid and vanillic acid, account for up to 12% of the total polyphenols [1,2,15].

These phenolic compounds are able to inhibit the enzymes involved in oxidative stress, scavenge free radicals or eliminate reactive oxygen species (ROS) responsible for the oxidation of biological matter and triggering aging and various diseases that progress with the inflammatory conditions [1,3]. They also stimulate the regeneration of other antioxidants (α-tocopherol) and endogenous antioxidant defense systems [3]. Oxidative stress is defined as an imbalance between oxidants and antioxidants to the detriment of antioxidants, resulting in abnormalities in oxo-reductive reactions and molecular damage [39]. It causes the formation of significant amounts of ROS, which include O_2_−, HO−, NO, RO− [3,39]. The most often described and synthesized radical is the superoxide anion (O_2_−), which has a moderate reactivity that allows it to interfere with cellular processes. It has the ability to convert to hydrogen peroxide (H_2_O_2_), which in turn can be recomposed into the highly reactive, hydroxyl radical (OH−). Hydroxyl radical, due to its high reactivity, can cause oxidative damage, which includes lipid peroxidation in membranes, oxidative modification of proteins and oxidative damage to DNA. Human skin is particularly exposed to ultraviolet and ionizing radiation, which are the main inducers of ROS formation [39]. Admittedly, it has enzymes and antioxidant compounds, but they are insufficient to inhibit oxidative damage, which is why additional support of the skin’s natural processes and components in neutralizing ROS is of high importance [39].

The evaluation of the antioxidant activity of *Vaccinium* plant extracts has been determined by various methods. Among them the results of the Folin-Ciocalteu test, the copper ion reducibility assay (CUPRAC), the ferric ion reducibility assay (FRAP), the DPPH (2,2-diphenyl-1-picrylhydrazyl) radical scavenging method, and the ABTS method were often described in the scientific literature [3].

The Folin-Ciocalteu method is the reference method for determining total polyphenol content (TPC). It is based on the oxidation of phenolic compounds present in the extract under the influence of a complex of phosphomolybdic and phosphtungstic acids in the presence of gallic acid as a standard with the formation of a blue-colored reaction product. The absorbance is measured at λ = 765 nm and is directly proportional to the TPC in the sample [3,9]. The DPPH method is a nonspecific method for determining antioxidant activity. The DPPH radical in solution shows a purple color with maximum absorbance at λ = 515 nm. When reacting with a radical scavenger, it captures its electrons which is accompanied by a change in the color of the solution to yellow. The result can be expressed as the percentage of scavenged radicals or by the IC_50_ index, that indicates the concentration of a tested sample that is necessary to scavenge 50% of a radical.

The ABTS test is used to measure the ability of the free radical scavengers to neutralize 2,2′-azinobis(3-ethylbenzthiazoline-6-sulfonic acid). The ABTS radical in solution exhibits a blue-green color with maximum absorbance at λ = 734 nm. The intensity of the color decreases with a decreasing absorbance and an increasing antioxidant activity [9]. The CUPRAC method is based on the spectrophotometric absorbance measurement of a colored complex that is formed in the reaction of copper (I) ions with bato-cuproin (2,9-dimethyl-4,7-diphenyl-1,10-phenanthroline) or neocuproin (2,9-dimethyl-1,10-phenanthroline). At last, the FRAP method, similarly to the CUPRAC method, involves the spectrophotometric measurement of the absorption of a coloured complex that is formed when TPTZ (ferro-2,4,6-tripyridyl-S-thiazine complex) reacts with an antioxidant [14].

The scientific literature shows several examples of studies conducted on different *Vaccinium* species that prove their strong antiradical or antioxidant properties. In the research of Urbonaviciene et al. who determined the antioxidant activity of *V. myrtillus* depending on the plants cultivation area plants from Norway, Finland, Latvia and Lithuania were considered. Using the Folin-Ciocalteu method, it was found that the samples from Norway showed the highest average TPC value of 791 mg/100 g dry weight. Plants collected in Lithuania were characterized by the lowest total content (546 mg/100 g dry weight), however the resulting average value of polyphenols was high as it was calculated as of 668.5 mg/100 g dry weight. A similar correlation was noted for the total anthocyanin content (TAC) in the performed HPLC-based analyses using cyanidin 3-glucoside (C3G) as the reference compound. The highest concentration of these important colorants was found in the samples from Norway (475.4 mg/100 g dry weight) and the lowest from Lithuania (233 mg/100 g dry weight), averaging 354.2 mg/100 g dry weight. Other tests used by the researchers differentiated the samples in a similar way—with the FRAP test—confirming the highest antioxidant activity for the Norwegian samples (50.6 µmol TE/g FW), and the lowest for Lithuanian ones (40.2 µmol TE/g FW), averaging 45.4 µmol TE/g, and with the ABTS assay in which the antioxidant activity ranged between 60.9 and 106 µmol TE/g FW (average 83.45 µmol TE/g FW). According to the authors the presence of significant quantities of polyphenols in the analyzed extracts determined the total antioxidant activity of the extract [18].

In other studies, Wang et al. evaluated the antioxidant activity of the oil-in-water nanoemulsions containing *Vaccinium vitis-idaea* extract using the DPPH method. It was shown that the percentage of the radical solution inhibition for the 0.2 mmol/L blueberry extract was equal to 63.58 ± 2.95%, for its nano-emulsified extract 79.37 ± 3.18%, and for 0.1 mg/mL ascorbic acid was calculated as 85.43 ± 2.85%. In addition, the type of the formulation that was prepared was found to increase the bioavailability, stability and antioxidant capacity of the sample [19]. In this study 14 compounds from the groups of phenolic acids, flavonoids and terpenoids were identified in the leaf extracts by UHPLC-MS technique. Among them the most abundant components were apigenin 7-rhamnosyl-(1->2)-galacturonide followed by bis(4-ethylbenzylidene)sorbitol, and pinocembrin7-O-neohesperidoside-3-O-acetate. Also caffeic acid was found as the leading component of the extract. The authors do not exclude the role of anthocyanins in the total activity, however the introduced analytical conditions did not allow the authors for the identification of significant quantities in the analyzed samples.

Jo et al. examined the antioxidant activity of anthocyanins from *V. uliginosum* extract using DPPH and FRAP assays, and the extract’s free radical scavenging activity was determined as IC_50_. The radical uptake capacity using the DPPH reagent was calculated as 2.44 ± 0.09 mg/mL, while the FRAP activity was determined as 0.20 ± 0.00 mM [10]. In other studies Pascoa et al. determined the antioxidant activity of *V. corymbosum* leaves using Folin-Ciocalteu and ABTS tests. Depending on the cultivar tested, the TPC value was determined as 39.6–272.8 mg gallic acid/g dry leaf (the highest for Titan, and the lowest for Huron cultivar), and TAC (total anthocyanin content) value tested by the ABTS assay was 22.6–124.8 mM Trolox per gram of dried leaf (similarly to the TPC values, the highest potential was determined for Titan, whereas the lowest for Huron cultivars) [11].

Borowska et al. conducted a study comparing the antioxidant activity of *V. oxycoccos* and 5 varieties of *V. macrocarpon* (Ben Lear, Bergman, Early Richard, Pilgrim, Stevens). In their studies the average polyphenol content for the 5 varieties of *V. macrocarpon* determined by the Folin-Ciocalteu method was 296.3 mg/100 g fresh weight compared to *V. oxycoccos*, in which the number was calculated as 288.5 mg polyphenols/100 g fresh weight. The DPPH assay showed that *V. macrocarpon* varieties had a higher radical scavenging capacity with an average of 50.52 mmol TE/g fresh weight than *V. oxycoccos* with 39.6 mmol TE/g, while the ABTS test showed, the opposite situation, *V. oxycoccos* had a stronger antioxidant potential (16.4 µmol TE/g FW) than *V. macrocarpon* varieties (13.08 µmol TE/g FW). Next to the determination of the sums of secondary metabolites the Authors showed a marked concentration of resveratrol in the analyzed samples that may have impact on the total antioxidant activity of the varieties [40].

The scientific literature delivers more proofs on the antioxidant potential of *Vaccinium* species. The authors Toyama et al. examined the total polyphenol content, the total proanthocyanidin content and the antioxidant activity of *V. virgatum*. The first was determined by the Folin-Ciocalteu method and expressed in mg of gallic acid equivalents. The obtained value varied between 144 and 444 mg gallic acid equivalents/g DW. The total content of proanthocyanidins was up to 27.2 mg catechin/g DW, whereas the antioxidant activity estimated using the DPPH assay, showed the antioxidant activity potential as reaching the values of 2369 µmol TE/g DW. The conducted chromatographical analysis showed the presence of (−)- and (+)-epicatechin and procyanidin A2 in the highest quantity, whereas the GC-MS based compositional study revealed the presence of methyl palmitate, sinapyl alcohol, β-amyrin, methyl ester of 8,11-octadecadienoic acid, or methyl ester of linolenic acid as the leading components of volatile fraction [41]. This experiment showed a very strong antiradical characteristics of *Vaccinium virgatum* species that was due to a high content of proanthocyanidins, flavonoids and phenolic acids.

The antiradical and antioxidant studies often accompany other biological assays. Kim et al. conducted an experiment to show which part of *V. oldhamii* showed the greatest anti-inflammatory potential. Next to these studies, the authors also performed the DPPH test (using the extract’ concentrations of 25 µg/mL and 50 µg/mL) and the Folin-Ciocalteu assay (at 50 mg/mL) to compare the antiradical potential of the fruit, leaf and stem extracts. Both tests proved that stalk extracts showed the highest antioxidant potential and the fruits showed the weakest activity [42].

Another species—*V. dunalianum—*was tested by the authors Cheng et al. The researchers fractionated the methanolic extract into five fractions: petroleum ether (PF), chloroform (CF), ethyl acetate (EF), n-butanol (BF), and water (WF). The activity was tested using FRAP, ABTS, and DPPH assays, and the values were expressed as nmol trolox equivalent (TE)/g of extract. The EF fraction showed the best antioxidant activity: FRAP (extract 0.91 ± 0.04 nmol TE/g), ABTS (extract 12.85 ± 0.23 nmol TE/g) and DPPH (extract 0.44 ± 0.03 nmol TE/g), whereas the PF fraction showed no activity in the ABTS and DPPH tests and the lowest in the FRAP test of 1.35 ± 0.14 nmol TE/g extract. These data confirm that the antiradical potential of the plant is related to the presence of the constituents of medium to high polarity. The antioxidant activity was also shown to be generally correlated with TPC and TFC (total flavonoid content) values, which for EF and PF were calculated as: 59.65 ± 0.93 mg GAE/g and 12.68 ± 0.37 mg GAE/g, and 23.42 ± 1.10 mg RE/g and 2.71 ± 0.09 mg RE/g, respectively. The TPC was measured in gallic acid equivalents using a 1 mg/mL solution, and the TFC was measured in rutin equivalents [43]. Goyali et al. investigated the oxidative capacity on another species, namely *V. angustifolium*. The total content of polyphenols in an 80% aqueous extract containing acetone and 0.2% formic acid in a 1:4 ratio was determined by the Folin-Ciocalteu method as gallic acid equivalents, while the proanthocyanidin (TPC) and total flavonoid (TFC) contents were determined using a spectrophotometric method as catechin equivalents. It was found that the TPC value ranged from 34.2 to 42.7 mg GAE/g FW, TFC from 12.7 to 22.3 mg CE/g FW, and PAC from 4.7 to 6.5 mg CE/g FW [44].

All studies mentioned above prove a strong antiradical potential of different *Vaccinium* species (see Table 5). These properties are certainly conditioned by the presence of flavonoids, anthocyanins and phenolic acids that are abundantly present in the samples. The measured antiradical potential exceeds the activity of gallic acid or Trolox that have been used by different researchers in the assays, which can highlight the value of *Vaccinium* plants in the process of scavenging free radicals, activating the antioxidant pathways and regenerating the radical scavenging capacity of the cellular systems.

The radical scavenging properties of different species of *Vaccinium* described above were the ground for the evaluation of their potential in different models. Every year an increasing number of studies on *Vaccinium* extracts in cell lines and in vivo are being published. The majority of them are related to the assessment of anticancer properties and their activity in cancer cell lines, however, some authors investigate the impact of *Vaccinium metabolites* on the physiology of cells, animals and humans.

The studies in the cells denote interesting properties of fermented berries. Ziemlewska et al. [45] conducted a study to assess the ability of extracts and ferments obtained by fermentation from *R. nigrum, A. melanocarpa* and *V. myrtillus* to protect cells against oxidative compounds, i.e., peroxide hydrogen. For this purpose, two types of human skin cells were used: keratinocytes (HaCaT) and fibroblasts (BJ) and *Saccharomyces cerevisiae* yeast (wild-type strains and sod1Δ deletion mutants). It was observed that all extracts and ferments have a cytoprotective effect by reducing the ROS level. It was shown that 0.15% *V. myrtillus* and *Ribes nigrum* ferments obtained after 20 days of fermentation (F20) inhibit the cell cycle. Increasing the ferment concentration to 0.3% increases growth inhibition, while increasing it to 0.6% increases the growth inhibition completely inhibits cell growth. A similar effect was observed using a 20-day-old *A. melanocarpa* ferment. The authors of the study performed the cytotoxicity analysis using the neutral red (NR) uptake test and the Alabama blue (AB) test. The first one involves the detection of live cells by uptake of the NR dye, which stains the lysosomes of living cells, while the second test allows for the assessment of cell viability by assessing the functioning of the mitochondrial respiratory chain. Fruit extracts from *R. nigrum*, *A. melanocarpa* and *V. myrtillus* and their kombucha ferments were used after 10 and 20 days of fermentation at concentrations of 30 and 300 µg/ml. There was no cytotoxic effect against HaCaT and BJ at all concentrations. A positive effect of F10 ferments on the metabolism and viability of both cell types was noted, while it was observed that the percentage of viability of cells treated with F10 is similar or higher than that of cells treated with extracts. It has been noticed that while extending the fermentation time to 20 days a decrease in the viability of skin cells occurs. In the report the authors list a wide range of compounds that were identified in *V. myrtillus* that can influence its beneficial properties. Among them flavonoids- glycosides of quercetine and myricetin, phenolic acids, catechins and anthocyanins were present on the fingerprint of the analyzed samples with chlorogenic acid, gallic acid and epigallocatechin gallate as the leading components freed from plant matrix during the fermentation process.

The scientific tests have shown that ferments can be of high value and may be characterized with a stronger antioxidant activity [45]. Another group of scientists—Marracino et al. assessed the antioxidant potential of *V. floribundum* before and after the fermentation process using the *Lactiplantibacillus plantarum* bacteria. This species of bacteria is classified as to lactic acid bacteria (LAB), which have the ability to induce pro-inflammatory cytokines, i.e., interferon-γ (IFN-γ), some interleukins (IL) (IL-1β, IL-6, IL-12), TNFα, and oxide nitrogen (NO). To assess the cytotoxicity of berry extracts, human umbilical vein cells (HUVEC) were exposed to 24-h exposure to *V. floribundum* (P) extracts and fermented *V. florinundum* (PF) solutions with concentrations ranging from 0.25 to 25 µg/mL. There was no reduction in cellular metabolism and therefore no cytotoxic effect at the tested concentrations. Using a chemiluminescent (CL) bioassay, a decrease in intracellular hydrogen peroxide production was noted. It turned out that fermented berries with *Lactiplantibacillus plantarum* reduced oxidative stress more effectively than unfermented berries. Moreover, the use of fermented berries was observed to promote the proliferation and synthesis of iNOS mRNA in RAW 264.7 murine macrophages. Fermentation was proven to increase the content of quercetin aglycone (up to 1680.0 ± 0.2 µg/g), which is present in FP but not in P [46].

A few publications showed a positive effect of *Vaccinium species* in animals, including mice or Drosophila models.

Authors Zhang and Dai conducted a study to analyze the anti-aging effect of anthocyanin-containing extracts from *V. vitis-idaea* on male *Drosophila melanogaster*, which is a model organism due to their short life cycle and numerous metabolic pathways that are similar to mammals. It turned out that the use of anthocyanin extracts from bilberry (BANC) at the concentrations of 2.5, 5.0 and 10.0 mg/ml extended the life of the flies by 9.16%, 11.90% and 6.88%, respectively, compared to the control sample in which BANC was not used. SOD activity among flies treated with BANC at a concentration of 5 mg/mL for 2 days and exposed to UV radiation for 30 minutes increased by 14.87%, respectively, compared to the group exposed only to UV radiation, while in flies lived for 7 days this correlation was 23.53%. As proved by the study the ROS content in the fat bodies of *D. melanogaster* decreased by 58.39% in the group treated with BANC extract at a concentration of 5 mg/ml and exposed to UV radiation for 30 minutes compared to the group exposed only to UV radiation. This means that BANCs can significantly alleviate the aging process of *D. melanogaster* males [47].

Jo et al. evaluated the photoprotective properties of *V. ulinosum* extract in hairless mice that were irradiated with ultraviolet B (UVB) radiation. The extract was standardized for the presence of anthocyanins and among the determined metabolites contained delphinidin-3-*O*-glucoside at the highest detected concentration (572.14 ± 73.03 µg/g) that is followed by cyanidin-3-*O-*glucoside, and malvidin-3-*O-*glucoside. In the study mice received an ethanolic extract from *V. lichinosum* orally 5 days a week, and were subjected to radiation 3 times per week. The total dose of absorbed UV radiation was 78 MED. The administered extract increased the expression of tissue inhibitor of metalloproteinase (TIMP) and antioxidant-related genes and decreased the expression of matrix metalloproteinase (MMP). Also, a visible reduction in the levels of p38 protein, c-Jun N-terminal kinase (JNK), inflammation-activated cytokines, and UVB-induced extracellular signal-regulated kinase (ERK) phosphorylation have been also reported [10].

Another interesting study was performed in healthy volunteers. Authors Tadić et al. introduced *V. myrtillus* leaf macerate and seed oil to an oil-in-water (O/W) cream to investigate the effects of these substances on the skin. This 30-day-long study involved 25 healthy volunteers (20 women and 5 men) aged 23.36 ± 0.64 years. It involved applying the cream to the palm of the right forearm twice a day. As a result, it has been reported that the cream containing extract of bilberry leaf isolates and oil from its seeds increased the hydration of the stratum corneum and reduced the TEWL (transepidermal water loss), which significantly improved the status and functioning of the skin. There was also no evidence of erythema occurring while using the cream, which proves that the ingredients are well tolerated by the skin [48]. The leading metabolites of the preparations determined in an HPLC study included among others: chlorogenic acid, isoquercetin, resveratrol, epicatechin, rutoside, and hyperoside as the leading metabolites.

Mechikova et al. conducted a study aiming at the understanding of *V. axillare* fruit extract effects on the antioxidant processes in vivo in a model of induced oxidative stress. The study involved 40 white male mice of the CBA line, which were divided into four groups: a group of intact animals, a control group, a ‘cisplatin’ group and a ‘cisplatin + blueberry’ group. The group of intact animals remained in the vivarium throughout the experiment and did not receive any substance. The control group received 0.9% sodium chloride solution orally at a dose of 10 mL/kg body weight for 10 days. The ‘cisplatin’ group received, similarly to the control group, 0.9% sodium chloride solution at a dose of 10 mL/kg body weight every day, and additionally, on the fifth day of the experiment, cisplatin was administered once intraperitoneally at a dose of 7.5 mg/kg body weight. The “cisplatin + blueberry” group received *V. axillare* extract at a dose of 10 mL/kg body weight every day, and on the fifth day, additional intraperitoneal cisplatin dose was administered (7.5 mg/kg body weight). On day 11 of the experiment, the animals were sacrificed and the left kidney was harvested. It was noticed that cisplatin injection caused a three-fold increase in free radical oxidation in mouse kidneys compared to controls and intact cells. The activation of lipid peroxidation processes in the kidney was also observed. In the cisplatin + blueberry group, the oxidative stress rates were higher than in the control and intact sample, but significantly lower than in the cisplatin trial. Production of activated oxygen metabolites in the cisplatin group + blueberry was 36% lower than in the cisplatin group confirming a marked role of the fruit in the antioxidant activity. Similarly, the measured content of lipid hydroxides was 40% lower. Resistance to peroxidation increased by 28% by the addition of the berry extract and the inhibition of protective systems by cisplatin decreased by 26% [49].

In another study, Wang et al. determined the antioxidant activity of *V. ashei* anthocyanins in healthy male C57BL/6 J mice that were divided into 3 groups depending on the dose of blueberry extract that was administered to them: 100, 400 or 800 mg/kg body weight. The animals were sacrificed at various time points (6 min, 30 min, 1, 2, 4, 8, or 12 h) and then plasma, intestines, liver, eyeball tissues and adipose tissue were collected to compare the effect of blueberry extract on the antioxidant processes in various tissues. It has been shown that the antioxidant activity of *V. aslei* extract is proportional to the administered concentration. The higher the concentration of the extract, the higher the T-AOC value (total antioxidant capacity), but the lower the MDA (malondialdehyde) level [50].

### 3.4. UV-Protecting Activity of Vaccinium Species

Ultraviolet (UV) radiation, a part of the sunlight spectrum, is one of the most critical risk factors in the initiation and development of several skin disorders, including hyperpigmentation, photoaging and skin cancer [4]. Solar UV radiation at the earth’s surface comprises approximately 90–99% UVA and 1–10% UVB as the UVC is almost completely absorbed by the ozone layer. UVB (280–320 nm) is less penetrating that UVA and reaches mainly the epidermal layer of the skin. UVB exposure has been implicated in the formation of erythema, accompanied with sunburn, and edema. UVA radiation (320–400 nm) can also induce DNA breakdown and the production of reactive oxygen species (ROS) that damage the retinal photoreceptor cells. ROS generated by UVA alter mitogen-activated protein kinase (MAPK) signal transduction pathways, such as p38 MAPK and c-Jun N-terminal kinase (JNK), and phosphatidylinositol 3-kinase (PI3K)/protein kinase B (Akt) signaling pathways, which play crucial roles in the regulation of UVA-induced cellular apoptosis and survival [5].

Anthocyanins, the most abundant active components of *Vaccinium* sp. fruits are known for their potential to prevent and reverse harmful effects of UV radiation [51]. Therefore the extracts from *Vacinium* fruits are potentially promising components of sun-protecting cosmeceuticals. Most of the published data conforming the UV-protective effects of *Vacinium* were done using the berries of two species: *V*. *uliginosum* and *V. myrtillus.*

Bae et al. evaluated the protective effects of anthocyanin-rich extract from bog blueberry (*Vaccinium uliginosum* L.) (ATH-BBe) in UVB-irradiated human dermal fibroblasts. ATH-BBe attenuated UVB-induced cytotoxic response, reactive oxygen species (ROS) generation and the resultant DNA damage responsible for activation of p53 and Bad proteins. Preincubation of the cells with ATH-BBe prior to UVB exposure markedly suppressed the changes characteristic for photoaging, including collagen degradation via blunting production of collagenolytic matrix metalloproteinases (MMP). Additionally, ATH-BBe enhanced procollagen expression at transcriptional levels. The signaling pathways involved in ATH-BBe protective action included blocking of the nuclear factor kappaB (NF-κB) activation and MAPK-signaling cascade. UVB radiation enhanced nuclear translocation of NF-κB, which was reversed by the treatment with ATH-BBe. The UVB exposure activated signal-regulating kinase-1 (ASK-1)-signaling cascades of JNK and p38 mitogen-activated protein kinase (p38 MAPK), whereas ATH-BBe hampered phosphorylation of c-Jun, p53, and signal transducers and activators of transcription-1 (STAT-1) linked to these MAPK signaling pathways. ATH-BBe also decreased UV-B induced-release of proinflammatory interleukin (IL)-6 and IL-8. Main anthocyanins detected in the ATH-BB extract were cyanidin-3-glucoside, petunidin-3-glucoside, malvidin-3-glucoside, and delphinidin-3-glucoside. Therefore, these bog bilberry anthocyanins may be used as active compounds protecting the skin from UVB-induced skin photoaging [52].

Pambianchi and co-workers evaluated the protective effects of *Vaccinium uliginosum* fruit extract (100 μg/mL) for preventing the oxidative, inflammatory, and structural damage induced by exposure of skin ex vivo explants to 200 mJ of UVA/UVB radiation. Topical application of studied extract on the explants for 24 h before UV exposure decreased the levels of UV-induced oxidative and inflammatory markers, including as 4-hydroxynonenal (4HNE), heme-oxygenase-1 (HO-1), cyclooxygenase-2 (COX2), and aryl hydrocarbon receptor (AhR). *V. uliginosum* extract treatment also counteracted the loss of structural proteins filaggrin and involucrin induced by UV radiation. All these effects suggest the protective effect of *V. uliginous* fruit extract on skin damage and premature aging caused by UV exposure [53].

Using hairless mice model, Jo et al. showed that the anthocyanin-enriched extract from *V. uliginosum* improved also the signs of UVB-induced photodamage following oral administration. *V. uliginosum* prevented epidermal thickness and collagen degradation, downregulated the expression of matrix metalloproteases MMP2, 3, 9 and hyaluronidase, increased the levels of TMP1 and TIMP3 inhibitors as well as collagen type I alpha 1 chain (COL1a1). Extract consumption also increased the levels of antioxidant enzymes in mouse skin: SOD, CAT and GPx, in comparison with control animals, and downregulated inflammatory cytokines and UVB-induced phosphorylation of extracellular signaling regulated kinase (ERK), as well as Jun N-terminal kinase (JNK) and p38 protein levels [10].

Svobododa et al. investigated the effects of a *V. myrtillus* fruit extract against UVA-induced skin changes in human keratinocytes HaCaT, a well establish in vitro model for studying UV-mediated skin damage. The extract used in this study contained 25% *w*/*w* anthocyanins and cyanidin and delphinidin derivatives were the most abundant components of this extract. HaCaT cells were pre-treated with *V. myrtillus* extract (5–100 mg/L) before or after irradiation with UVA (10–40 J/cm^2^). Pre-treatment (1 h) or post-treatment (4 h) of HaCaT with fruit extract resulted in significant reduction of UVA-induced damage. The extract was able to decrease the generation of UVA-stimulated ROS, with the percentage of inhibition, at the higher concentrations tested (50–100 mg/L) was around 55% in pre-treatment and around 50% in post-treatment. Both pre-treatment and post-treatment with the *V. myrtillus* fruit extract also inhibited UVA-caused lipid peroxidation, and inhibited UVA-induced glutathione depletion in HaCaT cells [54]. In another study by the same group phenolic fractions from fruit extract of *V. myrtillus* were also investigated using human keratinocytes HaCaT irradiated with UVB. One hour pretreatment of HaCaT cells with *V. myrtillus* polyphenols (5–50 mg/L) prior to UVB irradiation (100 or 200 mJ/cm^2^) reduced the breakage of DNA together with caspase-3 and caspase-9 activity. The extract also significantly decreased reactive oxygen and nitrogen species (RONS) generation and partially diminished the expression of pro-inflammatory IL-6. Increased HaCaT viability, decreased DNA damage, caspase activation and RONS generation were also observed when the of *V. myrtillus* extract was applied on the cells after UV-radiation [55].

Calò et al. also studied the effects of *V. myrtillus* extract on UV-induced damage in HaCaT keratinocytes. The extract used in this study contained 339.3 mg/100 g fresh weight of total polyphenols and 297.4 mg/100 g fw anthocyanins. HaCaT keratinocytes were pre-treated for 1 h with extract at 320 μg/mL and then irradiated with UVA (8–40 J/cm^2^) or UVB (0.008–0.72 J/cm^2^). As a result, the extract decreased the UVA- and UVB-induced oxidative stress, reducing UVA-induced ROS generation (but not UVB-induced ROS production), and lipid peroxidation caused by both UV rays, however the effect on this parameter was not statistically significant. The authors demonstrated the ability of the extract to significantly reduce UVA (16 J/cm^2^)-induced DNA damage, micronucleus formation after UVA (8 J/cm^2^), and UVB (0.008 J/cm^2^) irradiation. To summarize, in this study the *V. myrtillus* extract showed anti-apoptotic effects after UVA exposure, but it was inactive on UVB-induced apoptosis [56].

The harmful effect of UV radiation is also expressed in relation to the retina, which is why retinal cells are a good experimental model for testing UV-protective substances. Ogawa and co-workers investigated UVA-protective effects of bilberry (*Vaccinium myrtillus* L.) and lingonberry (*Vaccinium vitis-idaea*) commercial fruit extracts and their main constituents (cyanidin, delphinidin, malvidin, *trans*-resveratrol, and procyanidin) on culture murine photoreceptor cells 661W. Bilberry and lingonberry extracts (at 30 and 10 µg/mL) and constituents (10–30 µM) improved cell viability and suppressed ROS generation. Bilberry extract and cyanidin inhibited phosphorylation of p38 MAPK, and bilberry extract also inhibited phosphorylation of JNK induced by UVA, as analyzed using Western blotting. The lingonberry extract, trans-resveratrol, and procyanidin alleviated the reduction of phosphorylated Akt levels by UVA. Finally, a co-treatment with 30 µg/mL bilberry and 10 µg/mL lingonberry extracts followed by UVA treatment showed an additive protective effect on cellular viability [53].

Bucci and co-workers increased the UV-protective activity of *V. myrtillus* fruit extract by its encapsulation in elastic nanocarriers with 100 nm diameter, named “nanoberries”. Both the extract and nanoberries were shown to effectively decreased cytotoxicity induced by UVA and UVC exposure of HaCaT keratinocytes (nanoberries were more effective than the free extract in respect of UVA cytotoxicity). Interestingly, for UVB radiation experiments the protective effect was significant only for the nanoberries but not for the free extract [57]. The UV-protective action of *Vaccinium* spp. That were proved in the scientific literature are summarized in the Table 6.

### 3.5. Antimicrobial Properties

The rich composition of *Vaccinium* extracts in polyphenols conditions the antimicrobial activity of the extracts. In the scientific literature, we find numerous examples of studies confirming the antibacterial properties of this gender. The representatives were proved to be alternatives to antibiotics, also being active against antibiotic-resistant bacteria. The scientists constantly report the antimicrobial action of various berry species against a wide range of Gram-positive and Gram-negative bacterial strains [59], however, for the sake of this review article the studies on the influence of *Vaccinium* species on skin pathogens solely are a priority.

Among the key pathogenic microbes of the skin *Staphylococcus aureus*, *Malassezia* spp., *Propionibacterium acnes* should be listed as both commensals of the skin but also strains that can become pathogens under some conditions. Also, dermatological disorders can be affected by the presence of dermatophytes like *Trichophyton* that is known to deliver tinea pedis and onychomycosis, *Pseudomonas aeruginosa—*a Gram-negative resistant bacterium inducing folliculitis, green nail syndrome or toe web infections, *Candida* species causing a fungal candidiasis or rash, and *Corynebacterium minutissimum* that induces erythrasma [60].

The majority of the studies on *Vaccinium* species include the evaluation of their action towards *Staphylococcus aureus* that is associated with the development of soft tissues and skin infections, atopic dermatitis. Studies on this strain are particularly important as it is spread in more than 20% of global population as a commensal, however, due to the production of toxins and enzymes it is often causing the development of inflammatory conditions [61].

The study of Lian et al. [62], investigated the antimicrobial activity against this Gram-positive pathogen- strain ACTT25923—of different berry fruit extracts (blueberry, raspberry, cranberry, strawberry and acai berry). Among the studied fruits, the American cranberry—*Vaccinium macrocarpon* was found to be the most efficient and the application of its juice led to the 14.8% inhibition of bacterial culture growth (after 48 h), in comparison to 6.0% that was calculated for blueberry (*Vaccinium myrtillus*).

In another study the antimicrobial potential of the extracts from 12 Nordic berries that were enriched in polyphenols (with sugars removed on C18 SPE columns) were evaluated against *S. aureus* and *Candida albicans* [63]. *Vaccinium myrtillus* extract was found to be microbicide against both *S. aureus* cells. Importantly, the storage of the extracts, even if it was leading to a decreased content in polyphenols, did not influence the activity. The remaining extracts did not show activity or were not tested.

*Vaccinium corymbosum* fruit and leaf infusions and decoctions were proved to inhibit the growth of methicillin resistant and sensitive *S. aureus* at 12.5 mg/mL in the case of leaves and 50 mg/mL in fruit extracts that were rich in quercetin-3-glucoside, chlorogenic and caffeic acids [64]. The tested samples were also effective biofilm formation inhibitors, DNase and coagulase inhibitors.

There are a few results on the inhibitory properties of berries in the treatment of acne. According to Chae et al. [65], *Vaccinium oldhami* fruit extracts at the concentration of 200 ug/mL showed an inhibition zone of 16 mm in the case of *Propinionibacterium acne* and 13 mm for *Staphylococcus aureus*. Also, cranberry extract was an ingredient of a patented herbal mixture against the growth of *Propionibacterium acnes* (US7198807B2).

As expected, the type of extract affects the total antimicrobial activity. In the study of Sedbare et al. [16] who compared ethanolic and water extract of *V. macrocarpon* the latter extracts were found to be most active against *S. aureus*, most probably due to a higher phenolic content. Interestingly, in the case of *V. oxycoccos* dried berry extracts no statistically significant difference between the extracts was described. The leading metabolites determined in the analyzed extracts were ursolic acid, peonidin chloride, cyanidin chloride, oleanolic acid, ursolic acid, peonidin-3-galactoside, quercetine-3-galactoside, cyanidin-3-galactoside, and cyanidin-3-arabinoside.

The aforementioned activity of berries is mainly related to the presence of polyphenols that lead to the destabilization of cytoplasmatic membranes in bacteria, to the inhibition of microbial enzymes formation and also, due to the induced variations in the microbial metabolism. Some of the species are characterized by anti-adherence properties which disturb the colonization by bacterial strains [17]. Rich composition of berries in the compounds from the group of flavanols, containing catechins provide new applications of the fruits in the treatment of resistant bacteria, e.g., in the infections caused by methicillin-resistant *Staphylococcus aureus* [66]. High therapeutic efficacy is due to the pH of berry extracts and their constituents, which is an important issue especially in terms of the evoked effects towards the stability of microbial cell membranes. The active constituents of berries, when present in the undissociated forms, are able to pass through bacterial membranes which are negatively charged and interfere with enzymatic processes inside the cells as they dissociate in an elevated pH of bacterial leading to an acidification, disturbing the metabolism and increasing the effects of other anitmicrobial agents [17,67].

Within time some of the bacteria, including the Gram-negative ones, managed to develop cellular mechanisms of protection from the aforementioned interferences. Also, the cellular wall of Gram-negative strains contains lipopolysaccharides that make it less permeable, even if the acidic environment caused by the constituents of berries leads to the decomposition of the lipopolysaccharide layer in the Gram-negative bacteria and a cytoplasmatic leakage [68] Considering these facts, the scientific literature mentions some ininbitory activity of *Vaccinium* spp. against *Pseudomonas aeruginosa*, even if this action is weaker than in the case of Gram-positive bacteria.

According to Sedbare and co-investigators [16] who studied the antimicrobial potential of cranberries—*Vaccinium oxycoccos* and *V. macrocarpon*, next to a confirmed antimicrobial action against Gram-positive bacteria, like against *S. aureus,* or *Staphylococcus epidermidis,* both cranberries showed a weak activity against Gram-negative strains and among them against *Pseudomonas aeruginosa.* Among the tested samples water extracts from the powdered fruits showed a slightly higher activity in both species. Ilić et al. [69] mentioned the occurrence of a 19 +/− 0.8 mm inhibition zone in their studies on a cold pressed juice from *V. macrocarpon*, when tested against *P. aeruginosa.* According to the author, the juice was rich in phenolic compounds and hexoses, followed by organic acids that could all enhance the antimicrobial potential of the sample. Miljkovic and colleagues [70] determined the MIC and MBC values for the methanolic extract from the fruits of *Vaccinium myrtillus* against *P. aeruginosa.* In their studies the ratio of MIC/MBC was presented as 31.5/126.0 mg/mL. Along with the results of the antimicrobial activity the authors presented a list of nine anthocyanins, the derivatives of peonidin, malvidin, delphinidin, cyanidin and resveratrol that were determined in the tested sample.

The studies on the antibacterial activities of berries are supplemented by a few publications mentioning their yeast growth inhibitory action. *Candida albicans* was selected as a model for this action by Georgescu et al. [71] who investigated bilberry, black currant, gooseberry, red currant, raspberry, sea buckthorn, strawberry and sour cherry extracts. Their studies showed only a weak activity of the tested *Vaccinium myrtillus* species against this pathogen. The identified components of the bilberry were: quercetin, rutin, cinnamic acid, catechin, syringic acid, caffeic acid, resveratrol and ferulic acid. Table 7 summarizes the outcomes of antimicrobial properties of berries.

## 4. *Vaccinium* Species in Dermatology

### 4.1. Skin Cancer Chemoprevention

**Figure 2:**
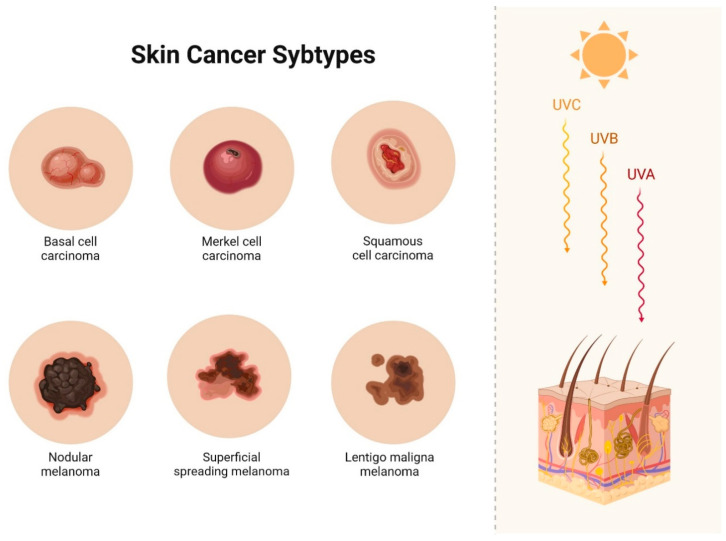
Skin cancer subtypes: basal cell carcinoma, merkel cell carcinoma, squamous cell carcinoma, nodular melanoma, superficial spreading melanoma, lentigo maligna melanoma. The figure was created by BioRender https://www.biorender.com/ (license to A.W., accessed on 20 June 2024).

Skin cancer ranks among the most devastating neoplasms of the current decade and is the fifth most common type of malignancy worldwide. Projections suggest that it may surpass heart disease as the leading cause of mortality and become the greatest obstacle to increasing life expectancy in the coming years [72]. Skin cancer, encompassing both malignant melanoma (nodular melanoma, superficial melanoma, lentigo maligna melanoma) and non-melanoma skin cancer (basal cell carcinoma, merkel cell carcinoma, squamous cell carcinoma), is the most prevalent malignancy among Caucasians (Figure 2) [73].

**Figure 3:**
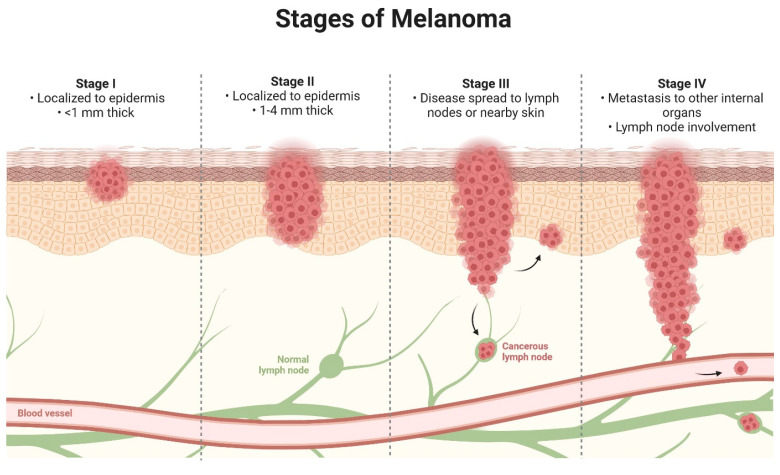
Four (I–IV) stages of melanoma—highly aggressive and fast-developing subtype of skin cancer. The figure was created by BioRender https://www.biorender.com/ (license to A.W. accessed on 12 June 2024).

Melanoma (Figure 3) is a highly aggressive and fast-developing subtype of skin cancer, affecting approximately 22 out of every 100,000 people. Treatment options for melanoma are limited, usually involving surgical resection of moles or chemotherapy. However, chemotherapy relates to long-term adverse effects. Recent advancements have improved survival rates using targeted small-molecule inhibitors and antibody-based immunotherapies [74].

In recent years, there has been a drastic increase in cases of skin cancer, including melanoma. The pathophysiology of skin cancer is complex, involving multiple contributing factors. Over the past decades, numerous trials have evaluated the efficacy of different active agents in halting the progression of UV radiation damage. Proactive treatment strategies for skin cancer have been explored through the chemoprevention of cutaneous malignancies in many clinical trials. Chemoprevention involves using synthetic or natural agents (also from the *Vaccinium* species) to prevent or reverse the development of skin cancer [75].

Polyphenols from plants have been described to provide photoprotective effects against various factors contributing to skin cancer. 1% acetic acid: methanol fraction of *Vaccinium macrocarpon* extract inhibited SK-MEL-5 (HTB-70 ™) melanoma cell line isolated from skin tissue obtained from a 24-year-old, White, female malignant melanoma patient with IC_50_ 147 mg/L [76]. Another study demonstrated that bilberry anthocyanins from *Vaccinium myrtillus* induced apoptosis of B16-F10 epithelial-like cells that were isolated from skin tissue of a C57BL/6J mouse with melanoma through activation of the mitochondrial pathway induced by reactive oxygen species. BAE also attenuated melanoma growth in vivo, as identified by TUNEL assays, hematoxylin-eosin staining, and Ki-67. Unfortunately, bilberry anthocyanin extract (BAE) diminished the effect of dacarbazine (DTIC), a chemotherapy medication used in the treatment of melanoma. Furthermore, western blotting analysis revealed increased in phospho-Akt expression after treatment with the combination of BAE and DTIC, indicating limited suppression of the PI3K/AKT pathway. Therefore, it is important to be cautious when using products enriched with BAE for melanoma patients undergoing treatment with DTIC [77].

### 4.2. Anti-Inflammatory Activity

**Figure 4:**
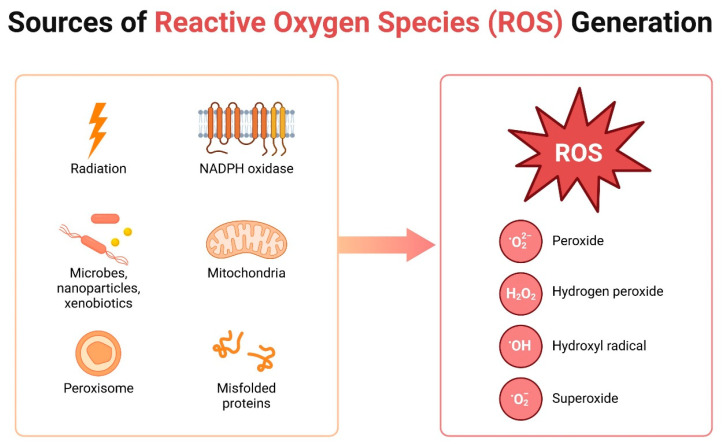
The sources of reactive oxygen species (ROS) generation: radiation; NADPH oxidase, microbes, nanoparticles, xenobiotics; mitochondria; peroxisome; misfolded proteins. ROS—reactive oxygen species. The figure was created by BioRender https://www.biorender.com/ (license to A.W. accessed on 12 June 2024).

Inflammation is an intensively studied cellular process associated with many diseases. The primary factor responsible for the exacerbation of chronic inflammation is oxidative stress, which arises from the increased production of free radicals, and an imbalance between the generation of reactive oxygen species (ROS) and their removal. An excessive amount of ROS induces oxidative damage to proteins, lipids, and DNA, which can subsequently lead to the loss of their cellular function (Figure 4) [78].

The lifestyle of modern society contributes to an increasing deficit of natural antioxidants. For this reason, understanding the molecular mechanisms underlying lifestyle diseases is crucial. Despite the high mortality associated with these conditions, currently available pharmacological treatments are increasingly scrutinized due to their toxicity and ineffectiveness. Therefore, there is an urgent need to seek dietary antioxidants to maintain the balance of ROS production and elimination, thereby preventing numerous diseases [79]. For this reason, medicinal plants are currently considered beneficial due to their properties like good availability, low cost, and lack of side effects compared to their synthetic agents [80].

Berries are one of the richest sources of bioactive compounds that have beneficial effects on health. Among the most popular species of berries from the genus *Vaccinium* are bilberry (*Vaccinium myrtillus*), blueberry (*Vaccinium corymbosum*), and cranberry (*Vaccinium macrocarpon*). Lingonberry (*Vaccinium vitis-idaea*) is less common due to limited cultivation.

**Figure 5:**
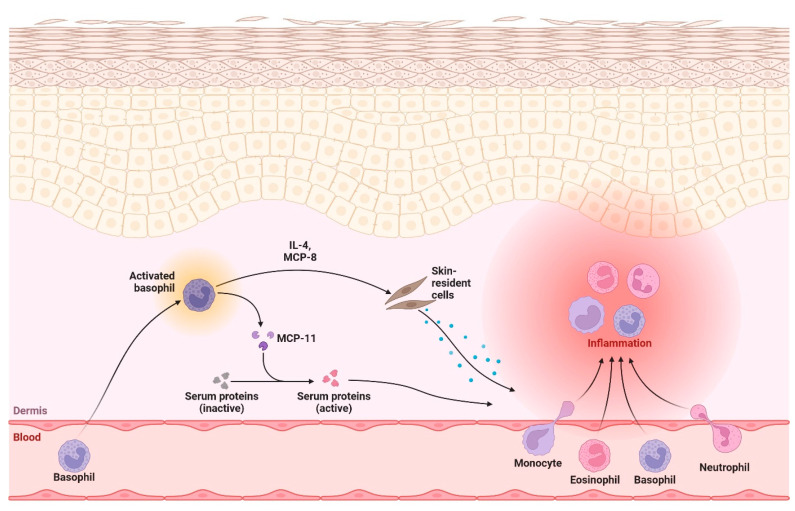
The basophil-mediated skin inflammation process. IL-4—interleukin 4, MCP-8—mast cell protease 8, MCP-11—mast cell protease 11. The figure was created by BioRender https://www.biorender.com/ (license to A.W., accessed on 12 June 2024).

The topical application of natural products has been studied for decades as a potential approach to prevent and cure different skin conditions, e.g., by reducing basophil-mediated skin inflammation (Figure 5).

Anthocyanins from bilberry (*V. myrtillus)* alleviate pruritus in a BALB/c mouse model of chronic allergic contact dermatitis. It has been shown that treatment with bilberon-25 (bilberry extract) significantly attenuated the 2,4,6-trinitro-1-chlorobenzene—induced increase in scratching behavior [81]. The effects of oral administration of a mixture of polyphenols and anthocyanins derived from *V. uliginosum* on 2,4-dinitrochlorobenzene—induced atopic dermatitis in NC/Nga mice was also determined. Oral administration of the mixture reduced the atopic-like skin symptoms as well as ear thickness and scratching behaviors, with concomitant reduction of the immunoglobulin E (IgE), immunoglobulin G1 (IgG1), T helper 2/1 (Th2/Th1) ratio, interleukin 4 (IL-4), interleukin 12 (IL-12), interleukin 13 (IL-13) and interferon gamma (IFN-γ) levels. [81] Blueberry extracts have shown efficacy in enhancing keratinocyte wound closure and normalizing the proliferation and migration responses that were previously altered by ozone (O3) exposure. Furthermore, pretreatment with blueberry extracts effectively prevented ozone-induced reactive oxygen species (ROS) production and inflammasome activation, as indicated by the formation of the NRLP1-ASC scaffold. This pretreatment also inhibited the transcription of key inflammasome components, including IL-18 and caspase 1 (CASP1) [82]. In turn, fermented blueberry and black rice extract containing *Lactobacillus plantarum* MG4221 treatment significantly reduced skin inflammation via downregulation of the mitogen-activated protein kinase (MAPK)/nuclear factor-κB (NF-κB) pathways and IL-1β, IL-6 and IL-8 levels in HaCaT cells [83].

Lingonberry (*V. vitis-idaea*) extract (50 mg/mL) reduced the levels of •OH and O•− radicals by 83% and 99%, respectively. The aqueous extract from lyophilized lingonberry fruit protected against oxidative stress associated with inflammation, reducing the production of ROS by 16%, 27%, and 31% at concentrations of 1, 2.5, and 5 mg/mL. Lingonberry extract also inhibited the expression of pro-inflammatory cytokines: IL-1β, IL-6, and monocyte chemoattractant protein 1 (MCP-1), but increased the anti-inflammatory cytokines expressions such as IL-10 and adiponectin. The extract demonstrated a high anti-inflammatory potential also in macrophage cell cultures by significantly downregulating the expression of pro-inflammatory mediators, including IL-1β, IL-6, MCP-1, tumor necrosis factor-alpha (TNF-α), cyclooxygenase-2 (COX-2), and inducible nitric oxide synthase (iNOS). Notably, the lingonberry extract exhibited a more potent inhibitory effect on iNOS expression and nitric oxide (NO) generation compared to budesonide, a glucocorticoid steroid known for its strong anti-inflammatory properties. Additionally, the lingonberry crude extract and a polyphenol-rich fraction demonstrated an anti-inflammatory activity at 100 ug/mL decreasing IL-1β, IL-6, COX-2 and iNOS gene expressions relative to the LPS-stimulated controls in RAW 264.7 macrophages. The anti-inflammatory effect of lingonberry juice was also demonstrated in spontaneously hypertensive rat in vivo model [84].

Clinical evidence suggests that cranberries (*V. macrocarpon*) and blueberries (*V. corymbosum*) alleviate oxidative stress, modulate inflammation, and alter neurotransmission [85]. The anti-inflammatory effects of bilberry (*V. myrtillus*) fruits and their extracts are due to their high anthocyanin content. Mirtoselect is a commercial extract standardized to contain 40% anthocyanins, which demonstrates complex anti-inflammatory activity in lipopolysaccharide-activated macrophages. This extract reduces the levels of pro-inflammatory cytokines, chemokines, and IL receptor expression induced by lipopolysaccharides almost to control levels. The response of THP-1 monocyte cells to inflammatory stimulation after treatment with anthocyanin-rich bilberry extract was more diverse. This extract decreased IFN-γ—induced activation of signaling proteins, cytokine secretion, and pro-inflammatory gene expression while increasing TNF-α-induced responses [86].

Studies have shown that the daily consumption of 500 g of blueberries by patients with gingivitis leads to a reduction in inflammatory cytokine levels and decreased bleeding on probing, which is a standard indicator of inflammation in dentistry. Another study conducted on patients with an elevated cardiovascular risk demonstrated that drinking 330 mL of bilberry juice daily for 4 weeks significantly reduced certain inflammatory markers in the blood, such as C-reactive protein, IL-6, and IL-15, compared to the control group. Individuals with metabolic syndrome consuming 400 g of fresh bilberries for eight weeks showed a reduction in several inflammatory parameters (C-reactive protein, IL-6, IL-12, and LPS). Additional evidence of the anti-inflammatory effects in individuals with metabolic syndrome was provided by a supplement containing purified anthocyanins from bilberries and blackcurrants. Inflammatory bowel disease, a functional gastrointestinal disorder, is often inadequately treated, leading to numerous adverse effects. There is a strong need to find an effective treatment. In vitro experiments have shown that bilberry fruit extract and isolated anthocyanins limit the inflammatory response of human colon epithelial cells. Both acute and chronic inflammation impact the development and progression of many diseases [87].

## 5. Conclusions and Future Perspectives

**Figure 6:**
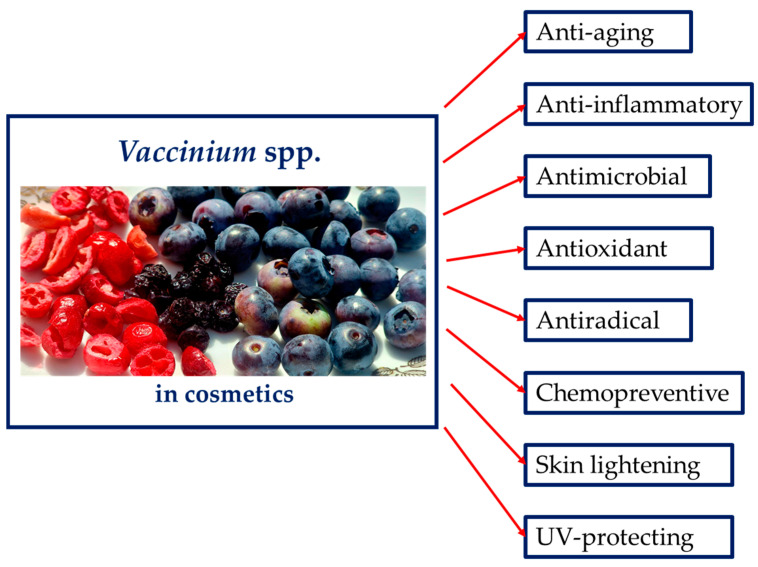
The cosmetic properties of *Vaccinium* species.

*Vaccinium* spp. represent shrubs with an exceptional composition. The high content of compounds from the most important classes of secondary metabolites of plant origin determines their vast and precious biological applications, from therapeutic effects in numerous diseases to valuable cosmetic properties (see Figure 6).

*Vaccinium* spp. are rich sources of anthocyanins, flavonoids, organic and phenolic acids, as well as tannins, the presence of which in preparations determines their beneficial properties, also for the skin. This article has collected the latest scientific reports showing a wide range of applications of berries in the cosmetics industry, emphasizing their important roles as ingredients with high antioxidant, anti-hyperpigmentation, anti-inflammatory, antibacterial, rejuvenating, and chemopreventive properties. Interestingly, extracts or active compounds from several *Vaccinium* species showed multidirectional activities, as summarized in Table 8. Extracts from *V. myrtillus* are distinguished by the most scientifically proven multidirectional cosmetic properties.

Most importantly, the review aimed to draw attention to plants of this genus and encourage further research on the introduction of new preparations with innovative applications in cosmetics, on the development of new effective formulations allowing a more efficient use of the potential of their ingredients, as well as on research on their bioavailability.

Among the most urgent directions of future studies on *Vaccinium* species are those related to the development of effective fermentation procedures and formulations that could lead to the recovery of higher quantities of active metabolites from plant matrix and a better bioavailability. Some studies on the application of fermented blueberry juice (*Vaccinium angustifolium* Aiton) have been already published giving way to new applications of these well-known plants. Fermentation performed with *Serratia vaccinii* showed new possibilities for the application of the fruits as anticellulite and anti-adipogenic agents which was proved in 3T3-L1 cells. The studied extracts and phenolics’ enriched fractions inhibited triglyceride accumulation via decreased p-AKT activation, increased p-AMPK and decreased the PPARγ activation to a similar extent as pure compounds catechol (CAT) and chlorogenic acid (CA) [88].

Another goal for the introduction of *Vaccinium*-based innovative cosmetics is the elaboration of nano-carrier forms with whose their penetration into the skin layers may be controlled, such as “nanoberries” containing fruit extract from *V. myrtillus*, developed by Bucci et al. and tested for their promising UV-protective activity and antioxidant properties [52]. *Vaccinium* fruit extracts might be also applied in other nanotechnology important for the cosmetic industry—“green synthesis” of metal nanoparticles. So far an antibacterial effect of ZnO nanoparticles synthesized using *Vaccinium* extract [89,90] has been established by the scientists and new formulation forms were elaborated, as it was described by Ogawa et al., who synthesized Alginate-, Carboxymethyl Cellulose-, and κ-Carrageenan-Based Microparticles as storage vehicles for cranberry extract [91]. Certainly, the preparation of modern application forms of *Vaccinium* extracts or metabolites is an important target for future development. Still the formulation studies using *Vaccinium* metabolites are scarce. The presented species are proved to exhibit interesting and important cosmetic values, however due to a high polarity of their major components and a limited penetration their presence in cosmetic products is limited.

## 6. Review Methodology

The data used for the preparation of the review article were delivered by different scientific databases such as ScienceDirect (https://www.sciencedirect.com/), PubMed/Medline (https://pubmed.ncbi.nlm.nih.gov/), Web of Science (https://www.webofscience.com/), Google Scholar (https://scholar.google.com/) and Scopus (https://www.elsevier.com/solutions/scopus) which were filtering the publications from the recent 20 years. To obtain information on the cosmetic properties of *Vaccinium* species presented in the Table 2 of the review, the CosIng database was used as it contains all registered species together with their cosmetic applications.

The following keywords were introduced to obtain the requested information: *Vaccinium* AND skin, anti-aging, tyrosinase, antimicrobial, antioxidant, UV, melanoma, melanin, inflammation, UV, and cosmetics (see Table 9 for the number of publications that were analysed). The manuscripts with clear indications of the cosmetic properties of *Vaccinium* species were taken into account and downloaded as full texts to extract the necessary information.

## Figures and Tables

**Table 1:** Phytochemical composition of *Vaccinium macrocarpon*.

Phytochemical/Class	References	Phytochemical/Class	References
Phenolic		Terpenes and terpenoids	
Phenolic acids		Monoterpenes	
Hydroxybenzoic acids		83. α-Terpinene	[18]
1. 1,2-Dihydroxybenzoic acid	[10,19]	84. (+)-α-Pinene	[18]
2. 2,4-Dihydroxybenzoic acid	[10,19]	85. (+)-3-Carene	[18]
3. 2,5-Dihydroxybenzoic acid	[18]	86. (−)-Camphene	[18]
4. 3-Hydroxybenzoic acid	[18]	87. (+)-Limonene	[18]
5. 4-Hydroxybenzoic acid	[18]	Monoterpenoids	
6. Benzoic Acid	[18]	88. Carvacrol	[18]
7. p-Anisic acid	[18]	89. (+)-Linalool	[18]
8. Gallic Acid	[18]	90. Nerol	[18]
9. Salicylic Acid	[18]	91. Monotropein	[18]
10. Syringic acid	[18]	92. 3-Methyl-3-buten-2-ol	[18]
11. Vanillic Acid	[18]	Triterpenoids	
Hydroxycinnamic acids		93. Oleanolic Acid	[18]
12. Caffeic Acid	[18]	94. Ursolic Acid	[11,18]
13. Chlorogenic Acid	[18]	95. 3-(p-Coumaroyl)ursolic acid	[18]
14. Cinnamic Acid	[18]	96. 3-O-p-hydroxycinnamoyl ursolic acid	[18]
15. o-Coumaric acid	[10,19]	Carotenoids	
16. p-Coumaric acid	[18]	97. Lutein	[11,18]
17. Ferulic acid	[18]	98. α-Carotene	[18]
18. cis-Ferulic acid	[10,19]	99. β-Carotene	[18]
19. Sinapinic acid	[18]	100. ζ-Carotene	[10,11,19]
Other phenolic acids		Phytosterols	
20. Ellagic Acid	[18]	101. β-Sitosterol	[18]
21. 4-Hydroxyphenylacetic acid	[18]	102. Sitogluside	[18]
Stilbenes		Other terpenoids	
22. Resveratrol	[18]	103. Isophorone	[18]
23. (Z)-resveratrol	[18]	Non-Phenolic organic acids	
Flavonoids		104. Aspartic Acid	[10,19]
Anthocyanidins		105. Citric Acid	[18]
24. Cyanidin	[18]	106. Fumaric Acid	[18]
25. Cyanidin 3-arabinoside	[18]	107. Glutamic Acid	[10,19]
26. Cyanidin 3-galactoside	[18]	108. Isocitric acid	[18]
27. Cyanidin 3-glucoside (kuromanin)	[10,19]	109. Malic Acid	[18]
28. Pelargonidin 3-galactoside	[18]	110. Oxalic Acid	[18]
29. Peonidin	[18]	111. Parasorbic acid	[18]
30. Peonidin 3-arabinoside	[11,18]	112. Phthalic acid	[18]
31. Peonidin 3-galactoside	[10,11,19]	113. Quinic acid	[18]
32. peonidin 3-O-β-D-glucoside betaine	[18]	114. Shikimic acid	[18]
33. Peonidin 3-glucoside	[10,19]	115. Tartaric acid	[18]
Chalcones		116. trans-Aconitic acid	[18]
34. Phlorizin	[18]	Aminoacids	
Flavanols		117. Alanine	[10,19]
35. Cianidanol	[18]	118. Arginine	[10,19]
36. Cinnamtannin B1	[10,19]	119. Aspartic acid	[10,19]
37. Epicatechin	[11,18]	120. Cystine	[10,19]
38. Procyanidin A2	[10,19]	121. Glutamic acid	[10,19]
39. Procyanidin B2	[11,18]	122. Glycine	[10,19]
Flavanones		123. Histidine	[10,19]
40. Prunin	[18]	124. Isoleucine	[10,19]
Flavonols		125. Leucine	[10,19]
41. Astragalin	[18]	126. Lysine	[10,19]
42. Hyperoside	[18]	127. Methionine	[10,19]
43. Kaempferol	[18]	128. Proline	[10,19]
44. Myricetin 3-arabinoside	[18]	129. Serine	[10,19]
45. Myricetin 3-O-β-D-glucopyranoside	[18]	130. Threonine	[10,19]
46. Myricetin 3-O-β-L-galactopyranoside	[18]	131. Tryptophan	[10,19]
47. Myricetin 3-O-β-D-xylopyranoside	[18]	132. Tyrosine	[10,19]
48. Quercetin	[18]	Sugars	
49. Quercetin 3-xyloside	[18]	133. Sucrose	[10,19]
50. Quercetin 3-O-arabinoside (avicularin)	[18]	Fatty acids	
51. Isoquercetin	[18]	Fatty alcohols	
52. Quercitrin	[18]	134. 1-Octanol	[18]
53. Rhamnetin	[18]	135. 3,7-Dimethyl-2,6-octadien-1-ol	[18]
54. Isorhamnetin	[18]	136. 1-Decanol	[18]
55. Isorhamnetin 3-O-β-L-galactopyranoside	[18]	137. 1-Hexanol	[18]
Isoflavonoids		138. 3-Pentanol	[11]
56. Genistein	[10,19]	139. Nonan-1-ol	[18]
57. Isoflavone	[10,19]	140. 2-Methyl-3-buten-2-ol	[18]
Vitamins		141. (3R)-octan-3-ol	[18]
58. Ascorbic Acid (vit.C)	[18]	142. (S)-(+)-2-Pentanol	[18]
59. Nicotinic acid (vit.B3)	[10,19]	143. (S)-(+)-2-Octanol	[18]
60. Pantothenic Acid (vit.B5)	[10,19]	144. (3R)-heptan-3-ol	[18]
61. Pyridoxine (vit.B6)	[10,19]	145. Oct-1-en-3-ol	[18]
62. Retinol (vit.A1)	[10,19]	Fatty aldehydes	
63. Riboflavin (vit.B2)	[10,19]	146. 2,4-Heptadienal	[18]
64. Thiamine (vit.B1)	[10,19]	147. 2-Decenal	[18]
65. Vitamin K	[10,19]	148. 2-Heptenal	[18]
66. α-, β-, γ-, δ-Tocopherol (Vit. E)	[18]	149. 2-Nonenal	[18]
67. α-, β-, γ-, δ-Tocotrienol (Vit. E)	[18]	150. Decanal	[18]
Miscellaneous phytochemicals		151. Dodecanal	[18]
68. Benzaldehyde	[18]	152. Hepta-2,4-dienal	[18]
69. 2-Phenylphenol	[18]	153. Heptanal	[18]
70. Benzothiazole	[18]	154. Hexanal	[18]
71. Benzyl Benzoate	[18]	155. Octanal	[18]
72. Benzyl formate	[18]	156. Undecanal	[11]
73. Betaine	[10,19]	Oxygenated hydrocarbons	
74. Choline	[10,19]	157. 1,2-Butanedione	[18]
75. Cystine	[10,19]	158. 2-Octadecanone	[11]
76. Ethyl benzoate	[18]	159. 2-Octanone	[18]
77. Eugenol	[18]	160. 2-Pentadecanone	[18]
78. Indene	[18]	161. 2-Tridecanone	[18]
79. Methyl benzoate	[18]	162. 6-Methyl-5-hepten-2-one	[18]
80. Phenol	[18]	Fatty esters	
81. Phenylboronic acid	[11]	163. Ethyl butyrate	[18]
82. Vacciniin	[18]	164. Monoethyl maleate	[18]

**Table 2:** *Vaccinium* spp. based cosmetic ingredients and their functions listed in the Cosmetic Ingredients Database CosIng [20].

Species	Ingredient (INCI)	Function in Cosmetic
*Vaccinium* *angustifolium*	VACCINIUM ANGUSTIFOLIUM FRUIT	Astringent, skin-conditioning
	VACCINIUM ANGUSTIFOLIUM FRUIT EXTRACT	Skin-conditioning, shooting
	VACCINIUM ANGUSTIFOLIUM FRUIT JUICE	Astringent, skin-conditioning
	LACTOBACILLUS/LEUCONOSTOC/BLUEBERRY FRUIT EXTRACT FERMENT FILTRATE	Humectant, skin-conditioning
	VACCINIUM ANGUSTIFOLIUM SEED	Abrasive
	VACCINIUM ANGUSTIFOLIUM LEAF EXTRACT	Skin-conditioning
*Vaccinium* *myrtillus*	VACCINIUM MYRTILLUS FRUIT JUICE	Skin-conditioning
	VACCINIUM MYRTILLUS FRUIT WATER	Skin-conditioning
	VACCINIUM MYRTILLUS FRUIT EXTRACT	Skin-conditioning
	VACCINIUM MYRTILLUS FRUIT/LEAF EXTRACT	Astringent, tonic, refreshing, skin-conditioning
	VACCINIUM MYRTILLUS LEAF EXTRACT	Astringent, skin-conditioning, nail-conditioning, hair-conditioning
	HYDROLYZED VACCINIUM MYRTILLUS LEAF EXTRACT	Skin-protecting
	VACCINIUM MYRTILLUS LEAF CELL EXTRACT	Skin-conditioning
	VACCINIUM MYRTILLUS BUD EXTRACT	Antioxidant
	VACCINIUM MYRTILLUS STEM EXTRACT	Antioxidant, astringent
	VACCINIUM MYRTILLUS SEED OIL	Skin-conditioning
	VACCINIUM MYRTILLUS SEED EXTRACT	Skin-conditioning
	VACCINIUM MYRTILLUS SEEDCAKE POWDER	Abrasive, skin-conditioning
	POLYESTER-22 (the polymer formed by the reaction of Octyldodecanol, dimer dilinoleyl alcohol, Succinic Acid and Vaccinium Myrtillus Seed Oil)	Skin-conditioning, emollient
*Vaccinium uliginosum*	VACCINIUM ULIGINOSUM BERRY EXTRACT	Skin-conditioning
*Vaccinium macrocarpon*	VACCINIUM MACROCARPON FRUIT	Skin-conditioning
	VACCINIUM MACROCARPON FRUIT EXTRACT	Astringent
	VACCINIUM MACROCARPON FRUIT JUICE	Skin-conditioning
	VACCINIUM MACROCARPON FRUIT WATER	Fragrance, perfuming
	VACCINIUM MACROCARPON FRUIT POWDER	Antioxidant
	LACTOBACILLUS/CRANBERRY FRUIT FERMENT EXTRACT	Antioxidant
	VACCINIUM MACROCARPON SEED	Abrasive
	VACCINIUM MACROCARPON SEED OIL	Skin-conditioning
	VACCINIUM MACROCARPON SEEDCAKE POWDER	Skin-conditioning
	VACCINIUM MACROCARPON SEED POWDER	Abrasive
	HYDROGENATED CRANBERRY SEED OIL	Skin-conditioning, emollient
	PEG-8 CRANBERRIATE (Fatty acids, cranberry (Vaccinium macrocarpon) seed oil, ethoxylated (8 mol EO average molar ratio)	Surfactant—Emulsifying
*Vaccinium dunalianum*	VACCINIUM DUNALIANUM LEAF EXTRACT	Skin-conditioning
*Vaccinium oxycoccos*	VACCINIUM OXYCOCCOS FRUIT EXTRACT	Skin-conditioning
	VACCINIUM OXYCOCCOS FRUIT WATER	Skin-conditioning
	VACCINIUM OXYCOCCOS SEED EXTRACT	Skin-conditioning
	VACCINIUM OXYCOCCOS SEEDCAKE	Skin-conditioning
*Vaccinium* *virgatum*	VACCINIUM VIRGATUM FRUIT JUICE	Humectant, skin-conditioning
	VACCINIUM VIRGATUM LEAF EXTRACT	Humectant, skin-conditioning
	VACCINIUM VIRGATUM STEM EXTRACT	Antioxidant
	VACCINIUM VIRGATUM CALLUS EXTRACT	Antioxidant, humectant, anti-sebum, hair-conditioning, skin-protecting
*Vaccinium* *oldhamii*	VACCINIUM OLDHAMII FRUIT EXTRACT	Skin-conditioning
*Vaccinium* *vitis-idaea*	VACCINIUM VITIS-IDAEA FRUIT EXTRACT	Antioxidant
	VACCINIUM VITIS-IDAEA FRUIT JUICE	Skin-conditioning
	VACCINIUM VITIS-IDAEA FRUIT WATER	Fragrance, skin-conditioning
	SACCHAROMYCES/VACCINIUM VITIS-IDAEA FRUIT FERMENT EXTRACT	Bleaching, skin-conditioning
	HYDROLYZED VACCINIUM VITIS-IDAEA FRUIT	Skin-conditioning
	VACCINIUM VITIS-IDAEA LEAF EXTRACT	Astringent, skin-conditioning, tonic
	VACCINIUM VITIS-IDAEA LEAF PROTOPLASTS	Antioxidant, humectant, skin-conditioning
	VACCINIUM VITIS-IDAEA SEED OIL	Antioxidant, emollient, skin-conditioning, skin-protecting
	VACCINIUM VITIS-IDAEA SEEDCAKE POWDER	Abrasive, Exfoliating
*Vaccinium corymbosum*	VACCINIUM CORYMBOSUM FRUIT	Skin-conditioning
	VACCINIUM CORYMBOSUM FRUIT EXTRACT	Skin-conditioning
	VACCINIUM CORYMBOSUM FRUIT WATER	Skin-conditioning
	VACCINIUM CORYMBOSUM SEED	Abrasive, skin-conditioning
	VACCINIUM CORYMBOSUM SEED OIL	Antioxidant, skin-conditioning, emollient

**Table 3:** Studies on the skin lightening properties of *Vaccinium* sp. extracts and isolated compounds.

*Vaccinium* Species	Experimental Model	Results	Reference
*Vaccinium macrocarpon* fruit juice (lyophylised)	Tyrosinase inhibition	78% inhibition for 1 mg/mL IC_50_ = 0.1064 ± 0.03630 µg/mL	[23]
*Vaccinium myrtillus* fruit juice (lyophylised)	Tyrosinase inhibition	58% inhibition for 1 mg/mL; IC_50_ = 0.4814 ± 0.09839 µg/mL	
*Vaccinium myrtillus*	Tyrosinase inhibition	H_2_O extract 0.25 mg/mL ca. 10% inhibition Met extract 0.25 mg/mL ca. 25% inhibition Met-H_2_O extract 0.25 mg/mL: 20% inhibition Ace-H_2_O extract at 0.25% mg/mL ca. 45% inhibition (IC_50_ = 2.21 ± 0.09 mg/mL)	[24]
*Vaccinium corymbosum*	Tyrosinase inhibition	Met-H_2_O extract 0.25 mg/mL: 20% inhibition Ace-H_2_O extract at 0.25% mg/mL ca. 45% inhibition H_2_O and Met extract at 0.25 mg/mL—no inhibitory activity	
*Vaccinium bracteatum and isolated compound p-coumaric acid (PCA)*	Mushroom tyrosinase inhibition HEK239 cells expressing human tyrosinase MelanoDerm skin model with melanocytes	The extract at 80 µg/mL inhibited human TYR by ca. 50% but did not inhibit mushroom TYR PCA was identified as the most active inhibitor of human TYR PCA at 5 mM reduced the melanin content in MelanoDerm tissues by ca. 13%	[27]
*Subcritical water extract (SWE) and hot water extract (HWE) from V. dunalianum leaves*	Monophenolase and diphenolase inhibitory assay	Monophenolase inhibitory activity: SWE: IC_50_ = 11.81 ± 0.52 μg/mL HWE: IC_50_ = and 24.50 ± 1.78 μg/mL Kojic acid IC_50_ = 9.33 ± 0.66 μg/mL Diphenolase inhibitory activity: SWE: IC_50_ = 21.17 ± 1.83 μg/mL HWE IC_50_ = 86.98 ± 3.46 μg/mL kojic acid: IC_50_ = 76.63 ± 4.71 μg/mL	[28]
*6′-O-caffeoylarbutin isolated from aerial parts of Vaccinium dunalianum*	Zebrafish melanogenesis assay	IC_20_: 63.89 mM for 6′-*O*-caffeoylarbutin 244.6 mM for arbutin The concentration required to prevent melanin production in zebrafish 60 mM for 6′-*O*-caffeoylarbutin 100 mM for arbutin	[30]
Pterostilbene isolated from various *Vaccinium* berries	B16F10 melanogenesis assay Zebrafish melanogenesis assay Human skin tissue explants	Concentration of pterostilbene effectively inhibiting melanin formation: 3 mM in B16F10 and zebrafish melanogenesis assays 10 mM in human skin explant experiments ↓ melanocyte dendritic development ↓ melanosome transport ↓ cAMP/PKA/CREB signal pathway	[32]

**Table 4:** Anti-aging properties of *Vaccinium* spp.

*Vaccinium* Species	Experimental Model (Cell Line/Enzyme/ Animal etc.)	Dose of Extract/Purified Compound	Results	Reference
*Vaccinium* *angustifolium*	Type I bovine collagen	5% extract (water/propylene glycol 40%) from Cosmetochem	inhibition of the glycation process by 76%	[37]
	In vitro reconstructed skin (Cell line KPH84) Staining of β1 integrin		AGE decline	
	ELISA—MMP-1		Reduces the amount of MMP-1 induced by ribose in the medium of reconstructed skin	
*Vaccinium* *myrtillus*	Hyaluronidase	0.05 mg/ml fruit extract (water/acetone 1:1)	Inhibitory activity was >90% IC_50_ [mg hydrogel] was 116.26 ± 26.65	
		0.5% extract evaporated from acetone-water mixtures (1:1 *v*/*v*) + 3.0% chitosan MMW		[24]
*Vaccinium* *uliginosum*	Hairless Mice (SKH-1)	Extract sample with ethanol, no exact dose	Anti-wrinkle effect	[10]
* Vaccinium macrocarpon *	Collagenase from *Porphyromonas gingivalis*	100 µg/mL AC-PAC—isolated from fruit	Greatest inhibitory effect—88.7% ± 8.5%	[35]
*Vaccinium* *vitis-idaea*	Women 35–50 years old	LAE double—water extract from lingonberries and amla fruits	Increased skin thickness by 120 µm Skin elasticity: Superficial—2x larger Deep—5x greater	[38]
*Vaccinium* *corymbosum*	Hyaluronidase	0.05 mg/mL fruit extract (water/acetone 1:1)	The inhibitory activity was >90%	[24]

**Table 5:** Antioxidant activity of the selected *Vaccinium* species.

Species	Antioxidant Activitity	Type of Assay	Reference
*V. myrtillus*	668.5 mg/100g	Folin-Ciocalteu	
	45.4 µmol TE */g	FRAP	[18]
	83.45 µmol TE/g	ABTS	
*V. vitis-idaea*	63.58% ± 2.95%	DPPH	[19]
*V. uliginosum*	IC_50_ = 2.44 ± 0.09 mg/mL	DPPH	
	IC_50_ = 0.20 ± 0.00 mM	FRAP	[10]
*V. corymbosum*	39.6–272.8 mg GAE */g	Folin-Ciocalteu	[11]
	22.6–124.8 mM TE/g	ABTS	
*V. macrocarpon*	296.3 mg/100 g	Folin-Ciocalteu	
	50.52 mmol TE/g	DPPH	[40]
	13.08 µmol TE/g	ABTS	
*V. oxycoccos*	288.5 mg/100 g	Folin-Ciocalteu	
	39.6 mmol TE/g	DPPH	[40]
	16.4 µmol TE/g	ABTS	
*V. virgatum*	144–444 mg GAE/g	Folin-Ciocalteu	[41]
	2369 µmol TE/g	DPPH	
*V. dunalifolium*	0.91 ± 0.04 nmol TE/g	FRAP	
	0.44 ± 0.03 nmol TE/g	DPPH	[43]
	12.85 ± 0.23 nmol TE/g	ABTS	
*V. angustifolium*	34.2–42.7 mg GAE/g	Folin-Ciocalteu	[44]

* GAE—gallic acid equivalent, TE—Trolox equivalents.

**Table 6:** UV-protective properties of *Vaccinium* sp. extracts and active compounds (*↑* - increase, *↓* - decrease).

*Vaccinium* Species	Experimental Model (Cell Line/Enzyme/ Animal etc.)	Dose of Extract/Purified Compound	Results	Reference
*Vaccinium uliginosum* (anthocyanin-rich fruit extract)	Human dermal fibroblasts	5 and 10 mg/L	↑ cell viability ↓ ROS generation following UVB exposure ↓ DNA damage ↓ collagen degradation ↑ procollagen synthesis ↓ NFκB nuclear translocation and MAPK activation ↓ IL-6 and IL-8 induced by UVB	[58]
*Vaccinium uliginosum (polyphenol-enriched fruit extract)*	Human ex vivo skin explants	100 µg/mL; topic application	↓ 4HNE ↓ HO-1 ↓ COX2 ↓ AhR ↓ COX2 ↑ structural proteins filaggrin and involucrin in response to UV exposure	[53]
*Vaccinium uliginosum (anthocyanin-enriched extract)*	Hairless mice (SKH-1, 4-week-old male)	Oral administration, no data on the dose	↓ epidermal thickness and collagen degradation ↓ MMP2, 3, 9 ↓ hyaluronidase ↑ TIMP1 and TIMP3 ↑ COL1a1 ↑ SOD1, CAT, GPx ↓ p-ERK, p-JNK, p-p38 following UV-exposure, in comparison with control mice (no extract administration)	[10]
*Vaccinium myrtillus* (anthocyanin-rich fruit extract)	HaCaT human keratinocytes	5–50 mg/L	↓ ROS ↓ lipid peroxidation ↓ glutathione depletion in response to UVA radiation following pre-treatment (1 h) or post-treatment (4 h)	[54]
*Vaccinium myrtillus* (polyphenolic-rich fruit extract)	HaCaT human keratinocytes	5–50 mg/L	↑ cell viability ↓ caspase-3 and 9 activation ↓ RONS ↓ DNA damage ↓ IL-6 release In response to UVB radiation following pre-treatment (1 h) or post-treatment (4 h)	[55]
*Vaccinium myrtillus* extract	HaCaT keratinocytes	320 µg/mL	↓ UVB-induced cytotoxicity, genotoxicity and lipid peroxidation ↓ UVA-induced genotoxicity, ROS and apoptosis	[56]
*Vaccinium myrtillus fruit extract (“nanoberries”)*	HaCaT keratinocytes	0.31–4.38 mg/mL	↑ viability in response to UVA, UVB and UVC exposure	[52]

**Table 7:** Antimicrobial potential of *Vaccinium* species.

Bacterial Strain	*Vaccinium* Species	Experimental Model	Dose of Extract/Purified Compound	Results	Reference
*Staphylococcus aureus* ACTT25923	*Vaccinium macrocarpon*, *Vaccinium myrtillus*	Inhibition of bacterial culture growth [%]	1 mL of juice	*V. macrocarpon*: 12.9 and 14.8% inhibition after 24 and 48 h, respectively *V. myrtillus*: 4.6 and 6.0%, respectively	[62]
*Staphylococcus aureus* E-045	Bilberry (*Vaccinium myrtillus*)	Liquid culture analysis	1 mg/mL	*V. myrtillus—*strong inhibition	[63]
*Staphylococcus aureus*	*Vaccinium corymbosum* fruit and leaf	MIC values	-	Leaf: 12.5 mg/mL Fruit: 50 mg/mL	[64]
*Propionibacterium acnes*	*Vaccinium oldhami*	Circular zone inhibition assay	200 µg/mL	16 mm clear zone	[65]
*Pseudomonas aeruginosa*	*Vaccinium macrocarpon*, *Vaccinium oxycoccos*	Circular zone inhibition assay	15 mg/mL	Slight activity	[16]
*Pseudomonas aeruginosa*	*Vaccinium macrocarpon*	Circular zone inhibition assay	60 µL of juice	19 +/− 0.8 mm inhibition zone	[69]
*Pseudomonas aeruginosa* ATCC 9027	*Vaccinium myrtillus*	MIC/MBC assay	-	31.5/126.0 mg/mL	[70]
*Candida albicans*	*Vaccinium myrtillus*	Circular zone inhibition assay	20 µL of undiluted hydroalcoholic extract (0.5 g in 10 mL)	Weak activity ca. 8 mm	[71]

**Table 8:** Summary of scientifically proven cosmetic properties of *Vaccinium* sp. (+ − confirmed properties).

*Vaccinium Species*	Skin-Lightening	Anti-Aging	Anti-Oxidant	UV-Protective	Anti-Microbial	Chemo-Preventive	Anti-Inflammatory
*V. macrocarpon*	+ [23]		+ [40]		+ [16,62,69]	+ [76]	+ [85]
*V. myrtillus*	+ [23,24]	+ [24]	+ [18]	+ [52,56,59,60]	+ [62,63,70]	+ [77]	+ [81,86]
*V. corymbosum*	+ [24]	+ [24]	+ [11]		+ [64]		+ [82,83,85,87]
*V. bracteatum*	+ [27]						
*V. dunalianum*	+ [28,30]		+ [43]				
*V. angustifolium*		+ [37]	+ [44]				
*V. uliginosum*		+ [10]	+ [10]	+ [10,53,58]			+ [81]
*V. vitis-idaea*		+ [38]	+ [19]				+ [84]
*V. oxycoccos*			+ [40]		+ [16]		
*V. virgatum*			+ [41]				
*V. florinundum*			+ [46]				
*V. axillare*			+ [49]				
*V. aslei*			+ [50]				
*V. oldhamii*					+ [65]		

**Table 9:** The number of publications found in the Pubmed database as a response to the introduced keywords (OP—original papers, RP—review papers).

	+Skin	+Tyrosinase or Melanin	+Anti-Aging	+Melanoma	+UV	+Skin + Inflammation	+Antioxidant	+Antimicrobial	Total Number
*Vaccinium angustifolium*	48 OP, 2 RP	8 OP	7 OP	2 OP	77 OP	4 OP	604 OP	165 OP	1981
*Vaccinium* *myrtillus*	23 OP, 1 RP	3 OP	1 OPa	2 OP	30 OP	3 OP	233 OP	57 OP	665
*Vaccinium macrocarpon*	8 OP, 2 RP	1 OP	2 OP	0 OP	22 OP	0 OP	261 OP	306 OP	1139
*Vaccinium uliginosum*	48 OP 2 RP	9 OP	7 OP	2 OP	81 OP	4 OP	598 OP	160 OP	1996
*Vaccinium dunalianum*	2 OP	4 OP	0 OP	0 OP	0 OP	0 OP	8 OP	3 OP	18
*Vaccinium* *oxycoccos*	0 OP	0 OP	0 OP	0 OP	1 OPa	0 OP	8 OP	8 OP	37
*Vaccinium* *virgatum*	46 OP, 2 RP	8 OP	7 OP	2 OP	76 OP	4 OP	590 OP	157 OP	1935
*Vaccinium* *oldhamii*	0 OP	0 OP	0 OP	0 OP	0 OP	0 OP	3 OP	1 OPa	10
*Vaccinium* *vitis-idaea*	3 OP	0 OP	2 OP	2 OP	12 OP	0 OP	74 OP	37 OP	249
*Vaccinium corymbosum*	53 OP 2 RP	9 OP	8 OP	2 OP	79 OP	4 OP	620 OP	162 OP	2052

## Data Availability

Not applicable.
